# Oncolytic adenovirus armed with cGAS activates STING pathway and enhances antitumor immunity in lung cancer with superior combined efficacy of PD-L1 therapy

**DOI:** 10.3389/fimmu.2026.1904206

**Published:** 2026-07-23

**Authors:** Qing-Wen Wang, Hua-Wei Xu, Yu-Sen Shi, Yi-Peng Zhang, Jie Jun, Dan-Ning Yue, Wei Zhao, Jia-Qiang Huang, Xiang-Lei Peng, Jie-Mei Yu, Jin-Sheng He, Yan-Peng Zheng, Yuan-Hui Fu

**Affiliations:** College of Life Sciences and Bioengineering, Beijing Jiaotong University, Beijing, China

**Keywords:** antitumor immunity, cGAS, cGAS-STING, NSCLC, oncolytic adenovirus

## Abstract

**Introduction:**

The extensive expression of STING in patients with non-small cell lung cancer (NSCLC) is closely associated with overall survival and other factors. Activation of the STING pathway can suppress NSCLC. However, the clinical translation of STING agonists remains hindered by challenges such as off-target effects, metabolic instability, and suboptimal pharmacokinetics.

**Methods:**

In this study, we engineered two oncolytic adenoviruses (OAds), OAd-HcGAS and OAd-McGAS, expressing human or murine cGAS, respectively, using an Ad5/3 chimeric adenovirus platform under regulation by the hTERT promoter to evaluate whether OVs carrying the cGAS gene are capable of specifically activating the STING pathway within tumors and enhancing the anti-tumor efficacy of OVs both *in vitro* and *in vivo*.

**Results:**

*In vitro*, OAd-HcGAS exhibited robust replication and potent cytolytic activity in tumor cells. It activated the STING–TBK1–IRF3 signaling axis, triggering a strong type I interferon (IFN-I) and pro-inflammatory cytokine response without compromising viral replication. In a murine Lewis lung carcinoma allograft model, intratumoral (i.t.) administration of OAd-McGAS led to substantial cGAS expression and consequential activation of the STING pathway. Moreover, the combination with anti-PD-L1 therapy resulted in tumor regression in over half of the cases. Notably, this armed oncolytic virus strategy enhanced the activation and infiltration of multiple immune cell populations.

**Discussion:**

Collectively, these findings establish cGAS-expressing oncolytic adenoviruses as a novel and effective therapeutic strategy for lung cancer treatment.

## Graphical Abstract

**Figure:**
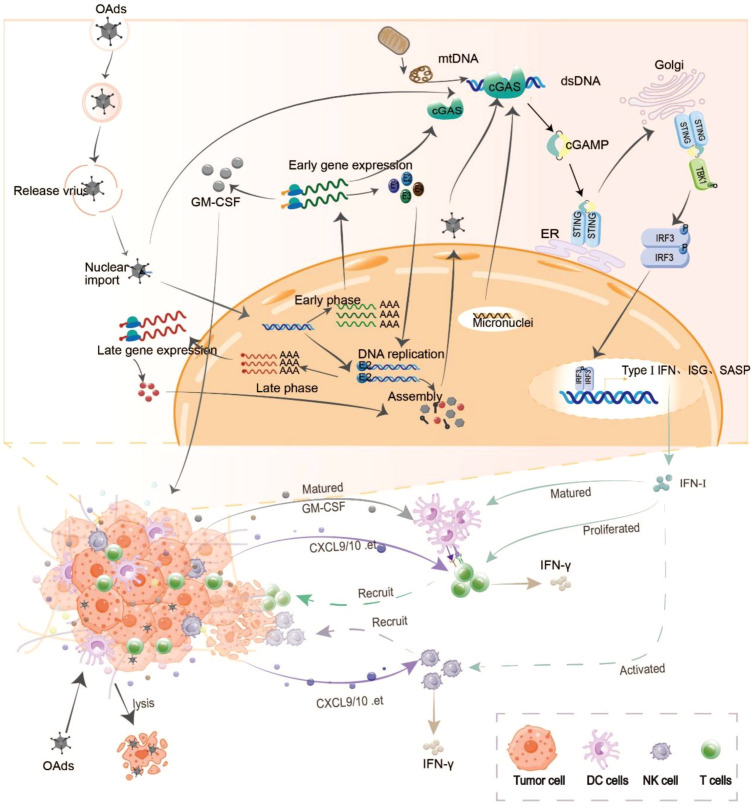
Viral replication and transgene expression and cancer treatment.

## Introduction

Although immune checkpoint inhibitors have demonstrated potential in the treatment of solid tumors, approximately 70% of patients with non-small cell lung cancer still fail to benefit due to limitations imposed by the tumor microenvironment and variations in PD-L1 expression (1, 2). In recent decades, oncolytic adenoviruses have emerged as a novel approach in lung cancer immunotherapy owing to their high gene transduction efficiency, controllable replication, and ability to induce immunogenic cell death (3). They enhance antitumor effects through the synergistic action of viral amplification and immune cell recruitment (4).

As a major cytoplasmic DNA-sensitive mechanism, the cGAS-STING pathway has attracted increasing attention for its capacity to stimulate and link innate and adaptive immune responses, thereby contributing to antitumor defense. The cytosolic sensor cGAS recognizes double-stranded DNA (dsDNA) and, upon binding, undergoes a conformational change that enables the synthesis of the second messenger cGAMP (5–8). Binding of cGAMP triggers STING oligomerization and recruitment of TBK1, which subsequently undergoes autophosphorylation and phosphorylates IRF3. Phosphorylated IRF3 then enables the nuclear translocation of IRF3 to drive type I interferon (IFN-I) expression, ultimately activating the innate immune system (9, 10). The cGAS–STING pathway is frequently downregulated across multiple cancer types and is strongly associated with unfavorable clinical outcomes. Studies in lung cancer indicate that targeted activation of this axis can enhance antitumor immunity (11, 12). For instance, one study demonstrated that combining HSV-1-based OVs with 2’3’-cGAMP enhanced systemic antitumor immunity and immune memory responses in both local and distant tumors through dendritic cell (DC) activation, without compromising viral replication—even in tumor cells with impaired STING-IFN signaling (13). Importantly, an intact STING pathway at the initial stage of tumorigenesis enables cGAS to detect self‐DNA fragments leaking from the nucleus, thereby suppressing tumor progression (14, 15). Although the STING pathway is frequently impaired in advanced tumor cells, this deficiency paradoxically creates a more favorable environment for OV replication (13).

STING activation can be stimulated by diverse factors, such as ionizing radiation, DDRi, and STING agonists (16). Although STING activation is critical for initiating innate immune responses, current STING agonists exhibit several limitations, including off-target activation, unstable metabolization, and suboptimal drug-like properties (17). Moreover, given that STING is generally expressed in both tumor and normal cells, the constitutive pharmacological activation of STING agonists can cause adverse immune reactions (18). These side effects significantly restrict their clinical application, highlighting the urgent need to develop strategies that achieve localized and tumor-specific activation of the STING pathway (19).

To enhance the restoration of endogenous anti-tumor immune responses while directly activating antitumor immunity, we generated two 5/3 chimeric oncolytic adenoviruses (OAds) expressing species-specific cGAS and GM-CSF, designated OAd-HcGAS (human) and OAd-McGAS (murine). We first evaluated their ability to replicate, express transgenes, and elicit direct antitumor effects in lung tumor cells. Our *in vitro* results demonstrated that OAd-HcGAS effectively infected and lysed human NCI-H226 cells while concurrently activating the STING pathway. Furthermore, in a murine Lewis lung carcinoma (LLC) allograft model, i.t. administration of OAd-McGAS not only initiated the STING pathway and induced IFN-I production, but also enhanced lymphocyte infiltration and elicited a significant abscopal effect. This study indicates that cGAS-expressing oncolytic adenoviruses represent a promising approach for lung cancer therapy.

## Results

### Construction of oncolytic viruses expressing human and murine cGAS

**Figure 1:**
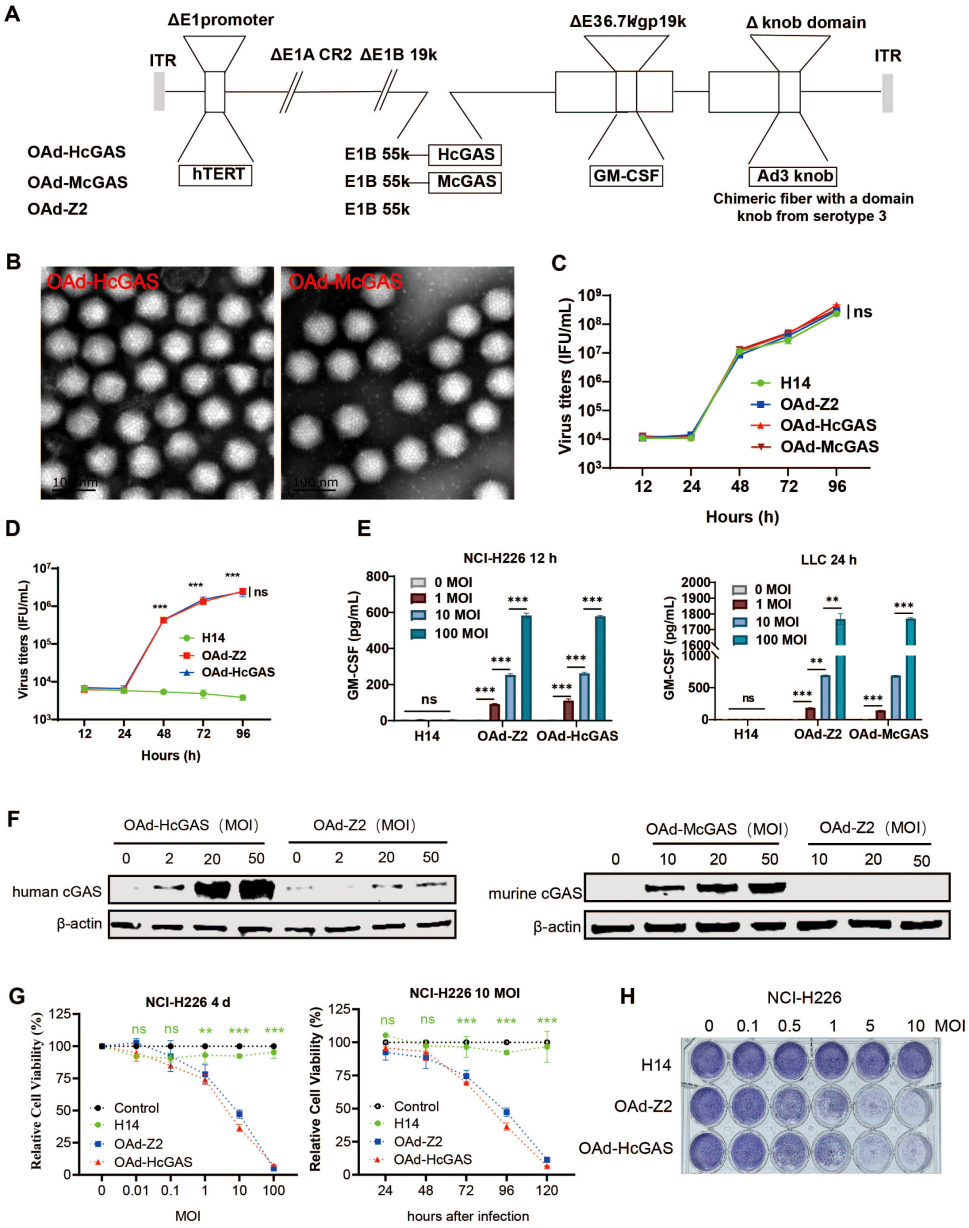
Functional verification of oncolytic adenovirus. **(A)** Schematic diagram of oncolytic adenovirus construction. OAd-HcGAS, recombinant adenovirus encoding human GM-CSF and human cGAS; OAd-McGAS, recombinant adenovirus encoding human GM-CSF and murine cGAS. **(B)** TEM of adenoviruses. **(C)** Viral replication capacity in HEK 293 cells infected at an MOI of 0.1 was measured using the plaque assay. **(D)** NCI-H226 cells were infected with recombinant adenovirus at an MOI of 0.5 and harvested at the indicated time points. Viral replication capacity determined by the plaque assay. **(E)** GM-CSF expression induced by OAd-HcGAS or OAd-McGAS was assessed in NCI-H226 and LLC cells. Cells were infected at various MOIs, and supernatants were collected 12 or 24 hours post-infection for GM-CSF quantification by ELISA. **(F)** cGAS protein expression in NCI-H226 and LLC cells after oncolytic adenovirus infection was evaluated via Western blotting. **(G)** The viability of NCI-H226 cells was evaluated using the CCK-8 assay at 24, 48, 72, 96, and 120-hours post-infection with OAds at varying MOIs, as well as at different time points following infection with a fixed dose of 10 MOIs. **(H)** Qualitative assessment of the cytocidal effect was conducted using the crystal violet assay at 96 hours post-infection across different MOI levels. Data ware shown as mean ± SD. **p < 0.01, ***p < 0.001, ns, no significant difference.

Analysis of the TIMER 3.0 database indicated broad expression of STING across both tumor and normal cells (**Supplementary Figure 1A**), highlighting the need for oncolytic viruses that selectively replicate in tumor cells to achieve tumor-restricted delivery of STING agonists. As illustrated in **Figure 1A**, OAd-HcGAS and OAd-McGAS were constructed on a chimeric OAd-Z2 bckbone, in which the E1B55k gene of the OAd-Z2 genome was fused with either human or murine cGAS via a P2A linker. The viruses were propagated through serial infection of HEK 293 cells, and transmission electron microscopy (TEM) confirmed typical adenovirus morphology (**Figure 1B**). Compared with OAd-Z2 and the replication-deficient adenovirus H14 in HEK 293 cells, both OAd-HcGAS and OAd-McGAS exhibited comparable replication kinetics (*p* > 0.05) (**Figure 1C**), indicating that the incorporation of cGAS and GM-CSF transgenes did not impair viral replication. Furthermore, ELISA verified GM-CSF expression (**Figure 1E**), and Western blotting confirmed cGAS expression in NCI-H226 cells infected with OAd-HcGAS and in LLC cells infected with OAd-McGAS (**Figure 1F**). These results collectively demonstrated the successful construction of novel recombinant oncolytic adenoviruses, OAd-HcGAS and OAd-McGAS, which express human and murine cGAS, respectively.

### OAd-HcGAS not only kills tumor cells but also activates the cGAS-STING pathway

To evaluate the cytotoxicity of OAd-HcGAS, various tumor cells were exposed to OAd-Z2 or OAd-HcGAS. CCK-8 assays demonstrated that the viability of all cell lines decreased in a time- and dose-dependent manner; Moreover, viability further decreased in a dose-responsive manner 96 h post-infection as viral titers increased (**Figures 1G**, **Supplementary Figures S1B–1E**). In parallel, crystal violet staining confirmed that both viruses nearly eliminated tumor cells at MOIs of 5 and 10 (**Figures 1H**, **Supplementary Figures S1B–1E**). These findings indicate that OAd-HcGAS exhibits oncolytic efficacy comparable to or greater than OAd-Z2 across multiple tumor cell lines, demonstrating that the induction of antiviral immune responses does not impair its intrinsic lytic capability.

**Figure 2:**
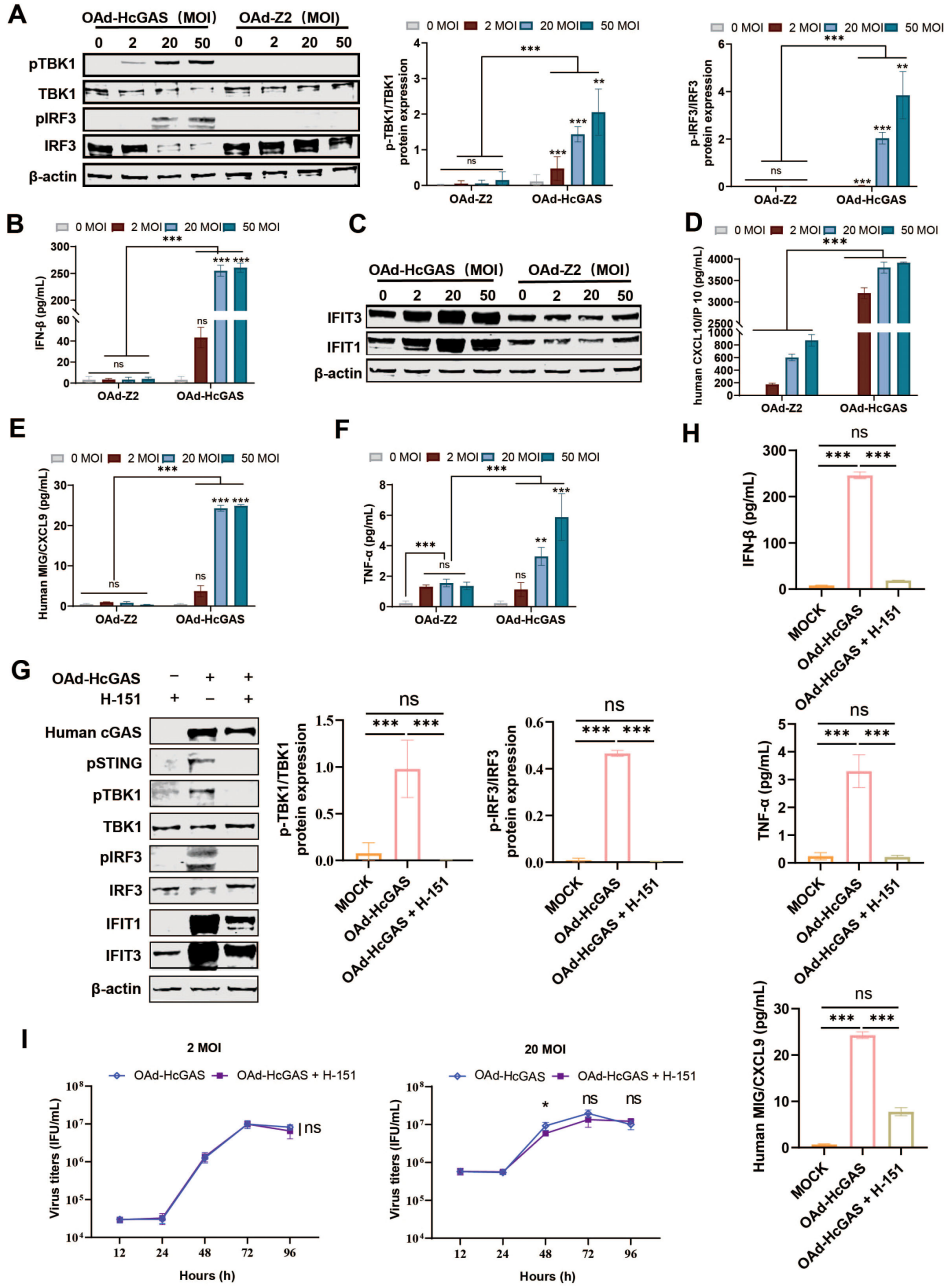
OAds activate the STING pathway in tumor cells. **(A)** Western blotting analysis of TBK1, pTBK1, IRF3, and pIRF3 in NCI-H226 cells infected with oncolytic virus at different MOIs for 48 hours. **(B)** ELISA quantification of IFN-β in cell supernatants. **(C)** Western blotting detection of IFIT1 and IFIT3 proteins. ELISA measurements of CXCL10 **(D)**, CXCL9 **(E)**, and TNF-α **(F)** in supernatants. **(G)** After STING pathway inhibition in NCI-H226 cells, infection with OAd-HcGAS (20 MOIs) was assessed by Western blotting for pSTING, TBK1, pTBK1, IRF3, pIRF3, IFIT1, and IFIT3. **(H)** ELISA analysis of IFN-β, CXCL9, and TNF-α secretion. **(I)** Viral replicative ability in NCI-H226 cells in the presence or absence of antagonist H-151. Data were shown as $\bar{X}\pm SD$, *p < 0.05, **p < 0.01, ***p < 0.001, ns, no significant difference.

Given that cytoplasmic dsDNA sensing by cGAS triggers STING signaling pathway (20, 21), we next examined its capacity to induce STING pathway activation in NCI-H226 cells. As anticipated, the expression levels of downstream proteins associated with the cGAS-STING pathway—such as phosphorylated TBK1, phosphorylated IRF3, IFN-β, proinflammatory factors (CXCL10, CXCL9, TNF-α, and interferon-stimulated genes (IFIT1, IFIT3)—were all significantly elevated (**Figures 2A–F**), while administration of STING inhibitors H-151 could suppress the activation of the STING pathway (**Figures 2G, H**). Efficient replication and proliferation of OVs in tumor cells, coupled with lysis of tumor cells by progeny virions, are central to their cytotoxic mechanisms. To assess the replication efficiency of OAd-HcGAS in NCI-H226 cells, viral titers were quantified at multiple time points following infection at an MOI of 0.1 using an immunoplaque assay. As shown in **Figure 1D**, OAd-HcGAS replicated robustly in NCI-H226 cells, with no statistically significant difference compared to the control virus OAd-Z2. Although the STING pathway typically suppresses viral replication as an antiviral defense mechanism, pretreatment with the H-151 inhibitor in this study revealed that STING activation induced by OAd-HcGAS did not significantly impair the replication efficiency of the virus in NCI-H226 cells (**Figure 2I**).

### OAd-HcGAS activates and recruits immune cells *in vitro*

**Figure 3:**
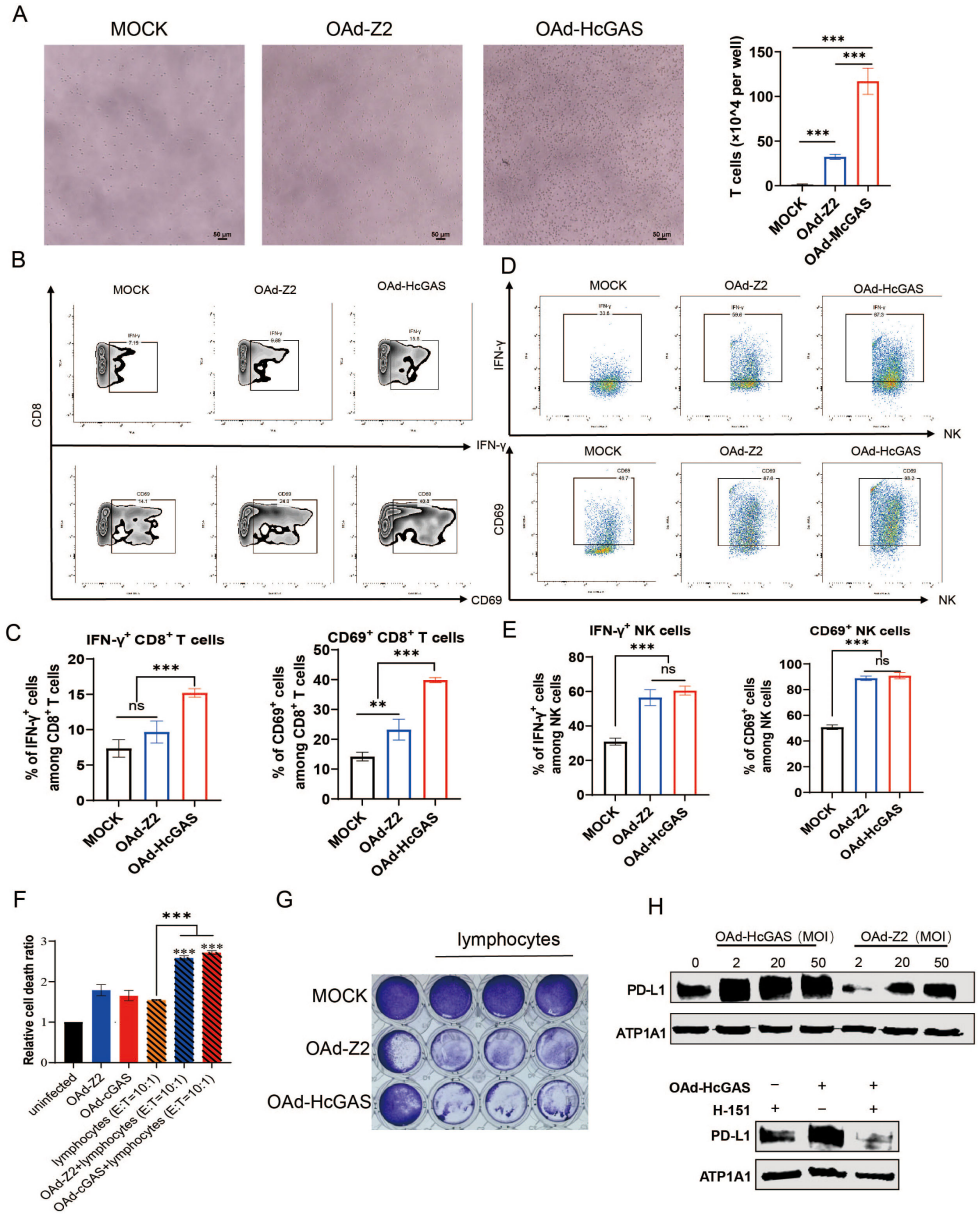
OAd-HcGAS can activate and recruit immune cells *in vitro*. **(A)** Lymphocyte recruitment and quantification in OAd-HcGAS-infected NCI-H226 cells. **(B-G)** NCI-H226 cells pre-infected with OAds were co-cultured with human PBMCs in a 1:10 ratio for 24 h before analysis by flow cytometry. Activation was measured by **(B, C)** expression of CD69 and IFN-γ on CD8 T cells or **(D, E)** expression of CD69 and IFN-γ on NK cells (n = 3 individual experiments). Cell death was measured by LDH cytotoxicity assay **(F)** and Crystal violet staining **(G)**. **(H)** PD-L1 proteins in NCI-H226 cells detected by Western blotting at 48 h after infection with OAd-HcGAS at different MOIs; Data were shown as $\bar{X}\pm SD$, **p < 0.01, ***p < 0.001, ns, no significant difference.

Infection with OAd-HcGAS markedly induced the secretion of CXCL10 and CXCL9 in NCI-H226 cells. To validate whether these secreted factors could promote lymphocyte migration, chemotaxis assays were performed using supernatants from OAd-HcGAS–infected NCI-H226 cells *in vitro*. As shown in **Figure 3A**, supernatant derived from OAd-HcGAS–infected NCI-H226 cells significantly enhanced lymphocyte migration into the lower chamber compared to both the OAd-Z2 control and the blank group. These results demonstrate that OAd-HcGAS infection stimulates tumor cells to secrete chemokines that facilitate lymphocyte recruitment to the tumor site.

To examine the immunostimulatory potential of OAd-HcGAS–infected NCI-H226 cells *in vitro*, a co-culture system was established combining these tumor cells with immune effector cells. As depicted in **Figures 3B–E**, co-culture with OAd-HcGAS–infected tumor cells led to a significant increase in CD69 expression on CD8^+^ T cells and NK cells (*p<* 0.05), a hallmark of early activation, and concurrently augmented IFN-γ secretion. The supernatants from the co-culture system were collected for LDH assay to quantify LDH released upon tumor cell lysis. Tumor cell mortality was significantly higher in co-culture systems containing OAds (*p* < 0.05, **Figure 3F**). Crystal violet staining showed that OAd-HcGAS-infected cells co-cultured with PBMCs exhibited markedly reduced staining intensity (**Figure 3G**). These findings suggest that OAds can effectively activate CD8^+^ T lymphocytes and NK cells to mediate cytotoxicity against tumor cells *in vitro*, with OAd-HcGAS exerting a stronger immune-stimulatory effect than OAd-Z2.

Additionally, consistent with previous reports (22, 23), our study also demonstrated that OAd-HcGAS–infected NCI-H226 cells significantly upregulated PD-L1 protein expression (**Figure 3H**), thereby providing a theoretical foundation for combining OAd-HcGAS with ICIs in subsequent therapeutic strategies.

### *In vivo* inhibitory effects of OAd-HcGAS on NCI-H226 lung cancer

**Figure 4:**
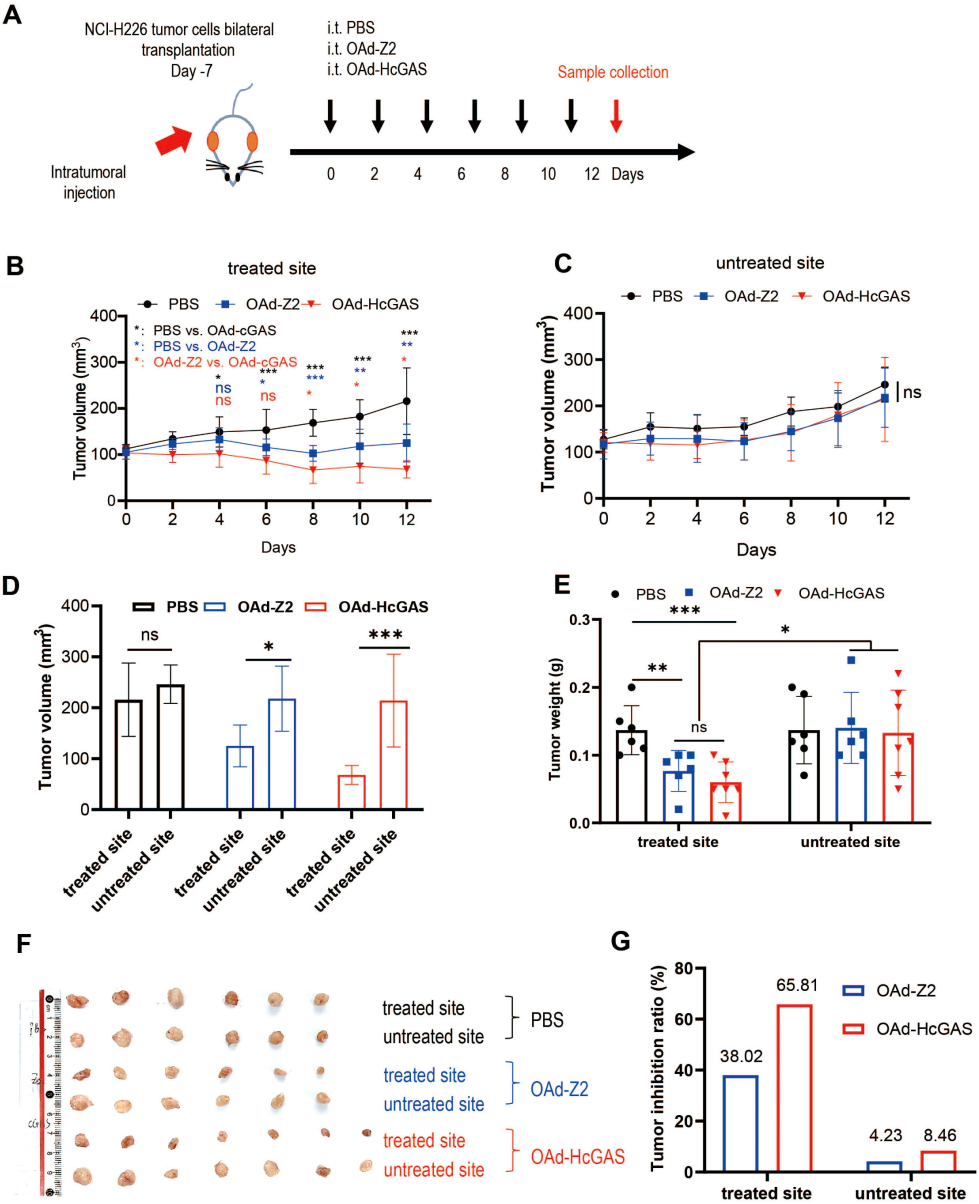
Inhibitory effect of OAd-HcGAS on NCI-H226 lung cancer. **(A)** Therapeutic scheme. In brief, one side was intratumorally administered with OAds (1×10^8^ PFU/tumor) every 2 days for 6 times in the bilateral subcutaneous NCI-H226 tumor model in BALB/c-nu immunodeficient mice, and the other side was untreated. (6 mice in the PBS group, 6 mice in the OAd-Z2 group, and 7 mice in the OAd-McGAS group) **(B)** Tumor growth curves of treated tumors; **(C)** Tumor growth curves of untreated tumors; **(D)** Tumor volume, **(E)** Tumor weight, **(F)** Representative tumor images and **(G)** Tumor inhibition rate on Day 12 of the treated and untreated sites. Data were shown as $\bar{X}\pm SD$, *p < 0.05, **p < 0.01, ***p < 0.001, ns, no significant difference.

To investigate the antitumor efficacy of OAd-HcGAS *in vivo*, a bilateral syngeneic model was established by implanting NCI-H226 human lung cancer cells into BALB/c-nu immunodeficient mice. To evaluate the antitumor efficacy of OAd-HcGAS, the oncolytic adenovirus was directly injected into transplanted tumors of 100–150 mm³. By Day 12, OAd-HcGAS achieved superior tumor suppression (68.07 mm³, 65.81% inhibition) compared to OAd-Z2 (125.06 mm³, 39.02%) and PBS (215.9 mm³) in treated tumors. No significant difference was observed in untreated tumors among groups (**Figures 4B, C, G**). Both OAd-Z2 and OAd-HcGAS resulted in significantly reduced tumor volumes on the treated side relative to the untreated side (*p<* 0.05), indicating localized therapeutic effects against NCI-H226 lung cancer.

### OAd-McGAS enhances antitumor efficacy and activates the cGAS-STING pathway *in vivo*

**Figure 5:**
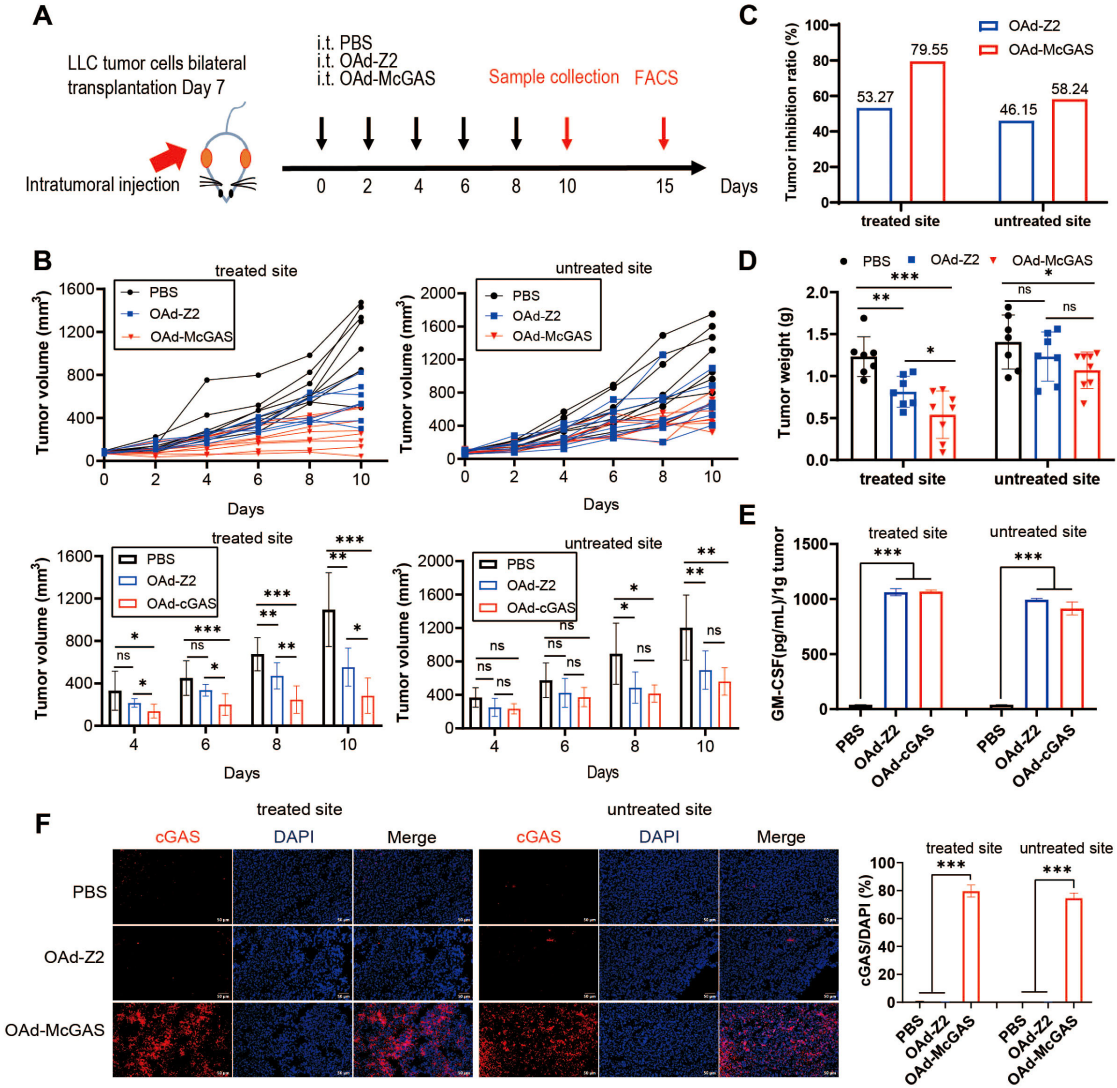
OAd-McGAS has a distant effect on tumor growth inhibition in LLC allograft tumors. **(A)** Therapeutic scheme. In brief, one side was intratumorally administered with OAds (1×10^8^ PFU/tumor) every 2 days for 5 times in the bilateral subcutaneous LLC tumor model in C57BL/6 mice, and the other side was untreated. (6 mice in the PBS group, 6 mice in the OAd-Z2 group, and 7 mice in the OAd-McGAS group) **(B)**The growth curve of each tumor volume on the treated and untreated sides; **(C)** The tumor inhibition ratio of treated and untreated sides; **(D)** The tumor weight of treated and untreated sides; **(E)** The expression of GM-CSF in treated and untreated tumor tissues determined by ELISA; **(F)** Immunofluorescence detection of cGAS protein expression (50 μm) and quantitative analysis of cGAS^+^ area percentage in three random fields from treated and untreated tumor tissues. Data were shown as $\bar{X}\pm SD$, *p < 0.05, **p < 0.01, ***p < 0.001, ns, no significant difference.

Bilateral LLC syngeneics were established in C57BL/6N mice to evaluate tumor growth inhibition after unilateral i.t. injection. As shown in **Figure 5B**, OAd-McGAS significantly reduced tumor volume on the treated side as early as Day 4 compared to OAd-Z2 (*p<* 0.05), whereas the reduction on the untreated side was modest and not statistically significant. By Day 10, tumor inhibition rates for OAd-McGAS reached 79.55% on the treated side and 58.24% on the untreated side (**Figure 5C**). Consistently, tumor weight measurements at Day 10 confirmed stronger suppression by OAd-McGAS, showing significant reductions compared to PBS on both sides (*p* < 0.05) and to OAd-Z2 on the treated side (*p* < 0.05; **Figure 5D**). These results indicate that OAd-McGAS, expressing murine cGAS, effectively inhibits the growth of LLC lung cancer syngeneics. An even more encouraging finding is that complete tumor regression was observed at the treated site on day 8 in the OAd-McGAS treatment group during repeated animal experiments (**Supplementary Figure 2F**).

**Figure 6:**
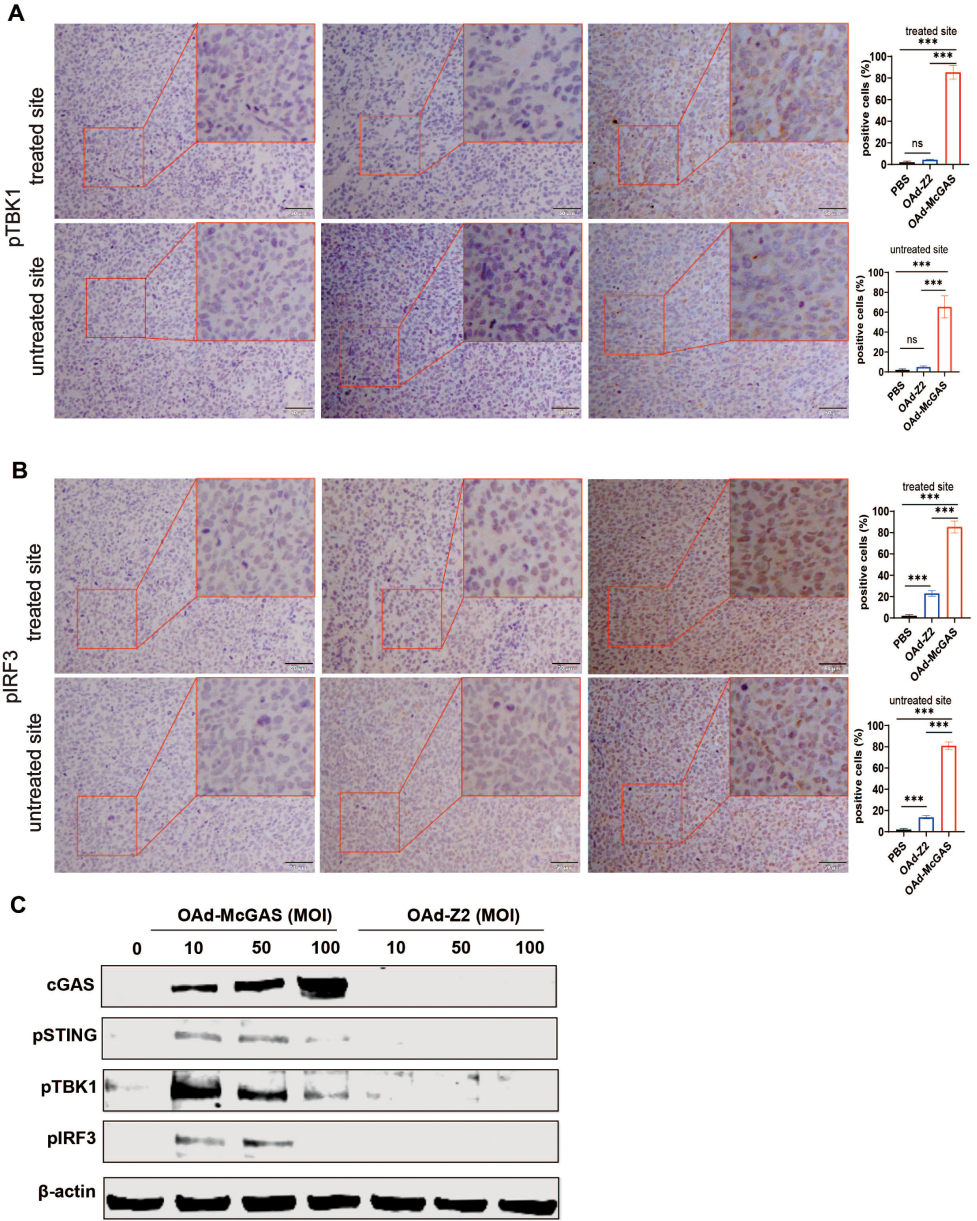
OAd-McGAS therapy can effectively activate STING pathway. Immunohistochemical staining was used to observe the status of pTBK1 (50 μm) in the tumor tissues on the treated side and the untreated side **(A)**, and the proportion of pTBK1 positive cells in the three random fields was statistically analyzed; **(B)** pIRF3 situation (50 μm) and statistics of the proportion of PIRF3-positive cells in three random fields; **(C)** Western blotting of pSTING, pTBK1, and pIRF3 in LLC cells infected with oncolytic virus at varying MOIs for 48 h. Data were shown as $\bar{X}\pm SD$, *p < 0.05, **p < 0.01, ***p < 0.001, ns, no significant difference.

Although human adenoviruses exhibit variable infectivity and replication in murine cells depending on serotype and cellular context, both OAd-Z2 and OAd-McGAS effectively infected in LLC cells, as evidenced by substantial cytopathic effects and robust expression of cGAS and GM-CSF at an MOI of 200 (**Supplementary Figure 2A**, **Figures 1E, F**). *In vivo* analyses further revealed elevated Hexon protein levels and increased viral DNA copies in both treated and untreated tumors from OAd-treated mice compared with PBS controls (*p<* 0.05; **Supplementary Figures 2B, C**). Hematoxylin and Eosin (H&E) staining revealed no significant tissue alterations or liver damage in mice treated with either OAd-Z2 or OAd-McGAS (**Supplementary Figure 2E**). Furthermore, overall body weight remained stable and gradually increased throughout the study (**Supplementary Figure 2D**), and i.t. administration of OAd-Z2 or OAd-McGAS did not induce severe physiological distress in the LLC transplanted tumor mouse model. Transgene expression of cGAS and GM-CSF was further confirmed by immunofluorescence and ELISA, respectively (*p<* 0.05; **Figures 5E, F**). As shown in **Figures 6A, B** and **Supplementary Figures 3A–G**, Immunohistochemical staining of tumor samples detected elevated expression levels of pTBK1, pIRF3, TBK1, IRF3 and downstream proteins, including CXCL10, IFIT1, IFIT3, TNF-α, and IFN-γ. Further supporting these findings, *in vitro* assays confirmed that OAd-McGAS enhanced the phosphorylation of STING, TBK1, and IRF3 (**Figure 6**). Interestingly, higher doses of OAd-HcGAS induced weaker STING pathway activation, a paradoxical effect that may be attributed to OAd-mediated inhibition of the STING signaling pathway (24).

### OAd-McGAS therapy efficiently increases tumor-infiltrating lymphocytes

**Figure 7:**
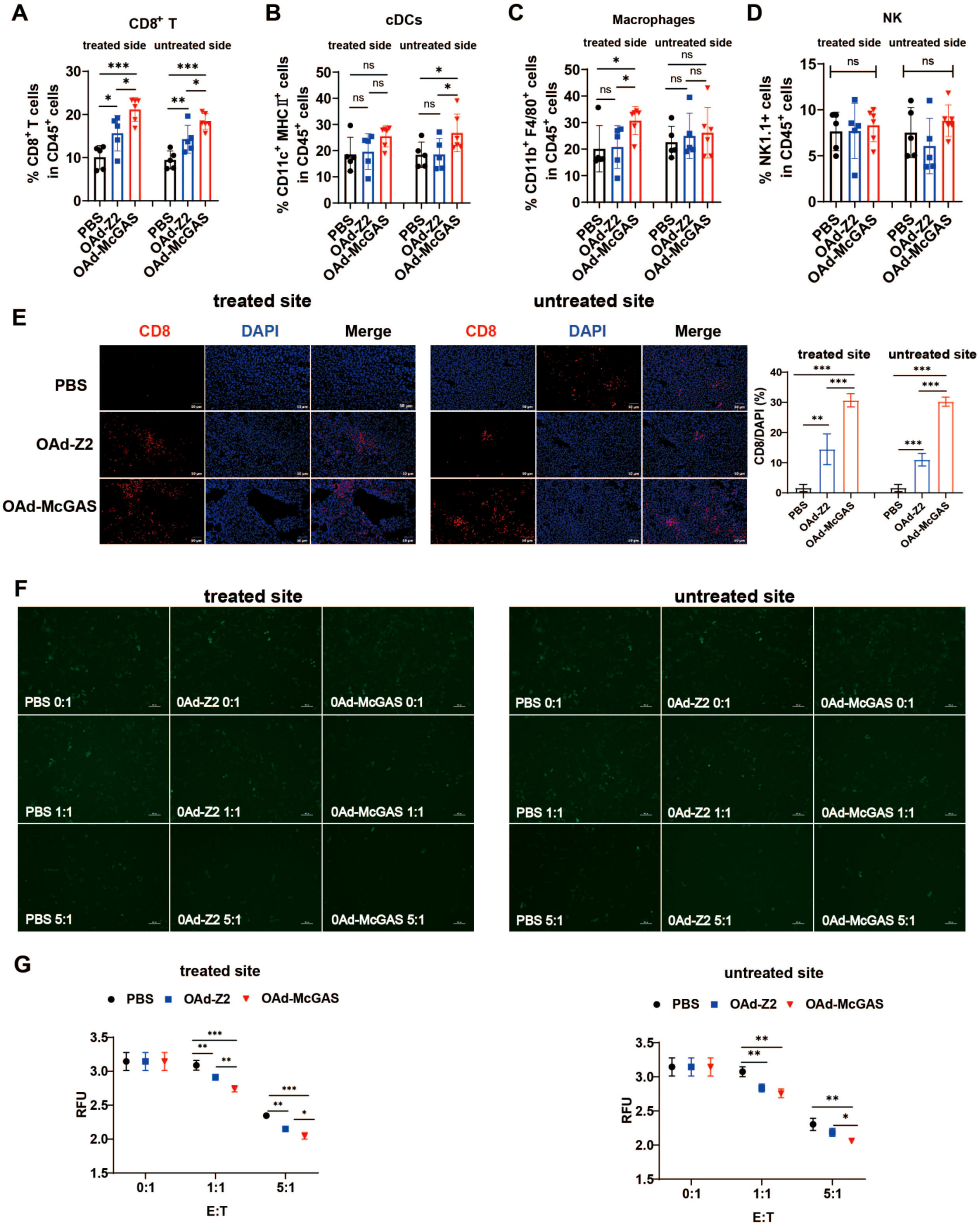
OAd-McGAS enhanced the tumoral recruitment of infiltrated lymphocytes, has obvious distance effect. LLC tumors were harvested on Day 15, digested enzymatically and stained for surface markers. FACS analysis of CD45+ CD8^+^ T cells **(A)**, CD45^+^CD3^-^CD11c^+^MHCII^+^ cDCs **(B)**, CD45^+^CD3^-^CD11b^+^F4/80^+^ Macrophages **(C)**, CD45^+^CD3^-^NK1.1^+^ NK cells **(D)**. **(E)** Representative figures for CD8^+^ T cells, in treated and untreated tumor tissues harvested from C57BL/6–10 day after the initiation of treatment were presented by IF and quantification analysis. TILs were separated from treated or untreated tumor and cocultured with target cells at E:T ratio of 0:1, 1:1 and 5:1, and the surviving target cells were confirmed, **(F)** Treated site and untreated site, observed by fluorescence microscope. **(G)** Treated site and untreated site, recorded by microplate reader. Data were shown as $\bar{X}\pm SD$, *p < 0.05, **p < 0.01, ***p < 0.001, ns, no significant difference.

As a potent cytoplasmic DNA sensor, cGAS initiates STING pathway activation in tumor cells and amplifies innate immune responses. To assess OAd-mediated immune responses, tumors were harvested on Day 15 post-treatment and analyzed by flow cytometry for tumor-infiltrating lymphocytes (TILs). As illustrated in **Figures 7A–C**, compared to the PBS and OAd-Z2 groups, the OAd-McGAS group showed an increase in the numbers of CD8^+^ T cells and tumor-associated macrophages (TAMs) within the tumor microenvironment on both the treated and untreated sides, whereas the increase in conventional dendritic cells (cDCs) did not reach statistical significance. No changes in NK cells were detected (**Figure 7D**). Immunofluorescence performed on Day 10 corroborated these findings, revealing higher CD8^+^ T cell density in both treated and untreated tumors (**Figure 7E**, *p* < 0.05). Further cytotoxicity assays revealed that TILs from OAd-McGAS–treated tumors effectively lysed LLC-EGFP target cells, as indicated by reduced green fluorescence and significantly lower RFU values at E:T co-culture (**Figures 7F, G**), demonstrating enhanced TIL killing activity that likely underlies its antitumor efficacy.

### The combination therapy of OAd-McGAS and anti-PD-L1 demonstrated potent antitumor efficacy *in vivo*

**Figure 8:**
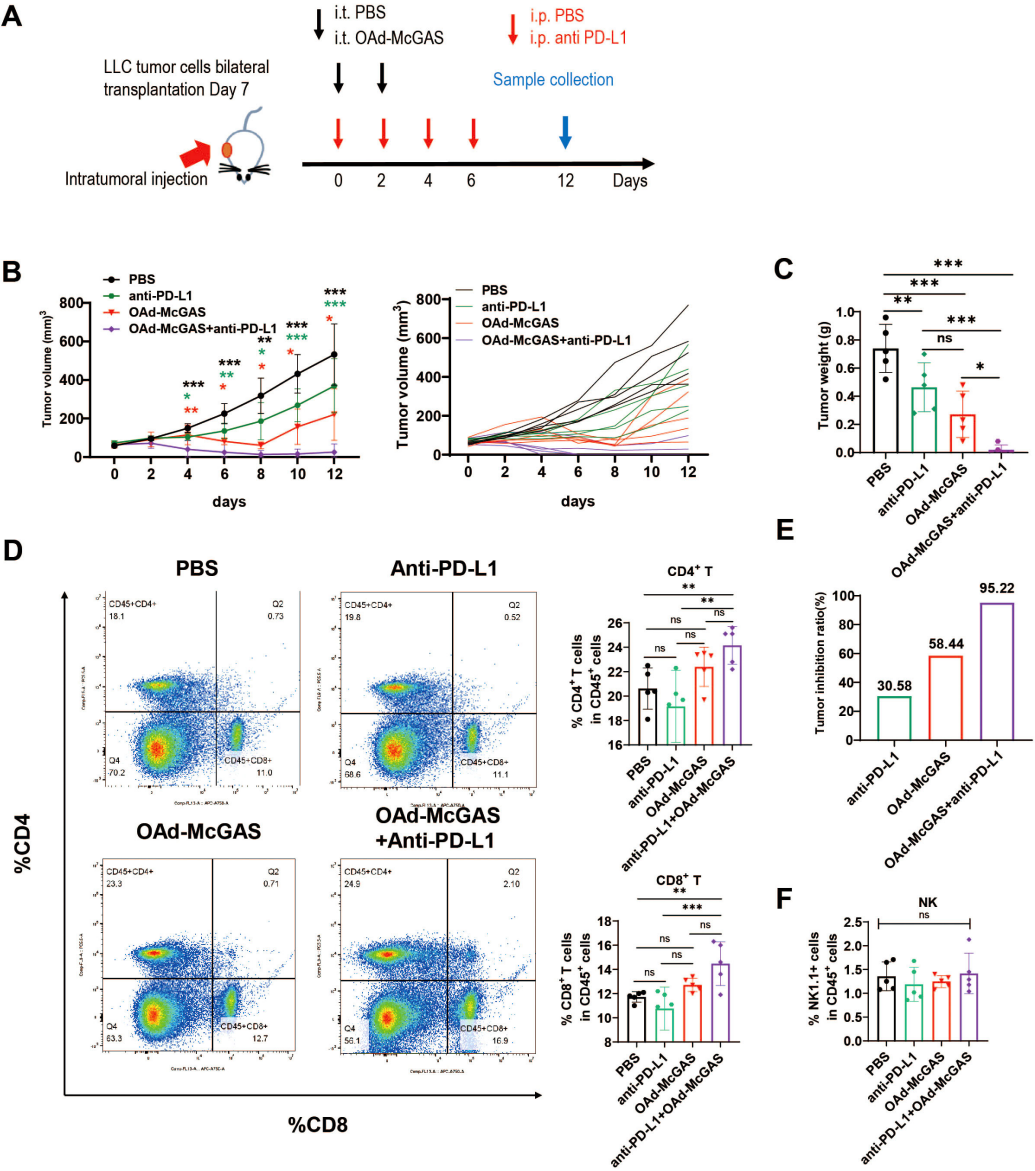
OAd-McGAS strongly improves the efficiency of anti-PD-L1 therapy. **(A)** Schematic diagram of the experimental setup for the combination therapy of recombinant adenovirus and PD-L1 antibody in LLC allograft model. OAd-McGAS were administered intratumorally (1×10^8^ PFU/tumor) every 2 days for a total of 2 doses, while PD-L1 antibody was administered intraperitoneally at a dose of 5 mg/kg every 2 days for a total of 4 doses. (n=5) **(B)** Tumor growth curves. **(C)** Tumor Weight. **(D)** Tumor inhibition ratio. FACS analysis of CD45^+^ CD4/8^+^ T cells **(E)** and CD45^+^ NK cells **(F)**. Data were shown as $\bar{X}\pm SD$, *p < 0.05, **p < 0.01, ***p < 0.001, ns, no significant difference.

As mentioned earlier, while OAd-McGAS is effective in treating LLC lung cancer, its therapeutic efficacy is limited and cannot completely cure the disease. We therefore further investigated whether combining OAd-McGAS with PD−L1 blockade could enhance anti−tumor efficacy. Therefore, a combination therapy experiment was conducted as illustrated in **Figure 8A**. The results demonstrated that OAd-McGAS significantly enhanced the therapeutic effect of the PD-L1 antibody. Among the five mice that received the combination treatment, three showed tumor regression (**Figures 8B, C**), with a tumor inhibition rate of 95.22% (**Figure 8E**). Flow cytometry was used to prove the capacity of the combination therapy to recruit immune cells. Combination therapy with OAd-McGAS significantly increased the proportions of CD4^+^ T cells and CD8^+^ T cells in the spleens of mice treated with PD-L1 antibody (**Figure 8D**, *p* < 0.05), while the proportion of NK cells remained unchanged (**Figure 8F**). The results demonstrated that OAd-McGAS significantly enhances the anti-tumor efficacy of the PD-L1 antibody.

## Discussion

The cGAS-STING signaling pathway is activated when cGAS senses cytoplasmic dsDNA in tumor cells, triggering tumor-specific CD8^+^ T cell activation and adaptive immune responses, accompanied by the release of proinflammatory cytokines and chemokines (10). This cascade promotes lymphocyte infiltration and modulates the immunosuppressive tumor microenvironment, positioning STING as a promising cancer therapy target. Despite its therapeutic potential, clinical application of STING agonists is hindered by limited efficacy, systemic inflammation, autoimmune-like side effects, and delivery difficulties (12). Given cGAS’s pivotal role in STING activation, we hypothesized that i.t. delivery of cGAS-armed OAds could locally activate STING signaling and exert anti-tumor effects. Building on our previous hTERT-promoter–regulated Ad5/3 chimeric OAd-Z2 expressing GM-CSF, we engineered OAd-HcGAS and OAd-McGAS by P2A-linking human or murine cGAS to the E1B55k locus of the OAd-Z2 genome. This strategy generated hTERT-regulated Ad5/3 chimeric oncolytic adenoviruses, OAd-HcGAS and OAd-McGAS, co-expressing GM-CSF and cGAS. *In vitro* experiments demonstrated that OAd-HcGAS not only effectively curbed neoplastic proliferation but also potently activated the STING signaling pathway. Furthermore, in a bilateral lung cancer model, OAds carrying the cGAS gene activated the STING signaling pathway, enhanced lymphocyte infiltration, and synergistic suppression of tumor growth on both treated and untreated sides, which collectively validates a pronounced “abscopal effect”.

*In vitro* experiments, we validated that OAd-HcGAS activated the STING pathway in NCI-H226 cells. A key concern was whether rapid vector clearance post-STING activation would impair viral production; however, comparative replication assays in HEK 293 and NCI-H226 cells showed comparable replication capacities among H14, OAd-Z2, OAd-HcGAS, and OAd-McGAS. In conclusion, OAds encoding cGAS effectively activate the STING signaling pathway without compromising viral replication efficiency. A study by Eric Lam et al. revealed that the cGAS/STING/TBK1/IRF3 signaling cascade is not directly targeted by viral anti-host strategies, and adenovirus-induced activation of the cGAS/STING DNA response does not significantly affect viral replication efficiency (25). Several mechanisms may explain this phenomenon. The adenoviral E1A protein can circumvent host antiviral responses by interacting with cellular proteins to inhibit viral DNA recognition, thereby suppressing the activities of cGAS and STING (24, 26). Moreover, as adenovirus replication occurs within the nucleus (27–30), cGAS primarily senses and binds exogenous or endogenous cytoplasmic dsDNA (31). To avoid detection by cGAS, adenoviruses encapsulate their DNA within protein coats. During OAd construction, the E1A CR2 region, which binds to the RB protein, was deleted. Consequently, E1A is not inhibited by interferon, allowing viral replication to proceed unaffected even when OAds activate the IFN-I system. Furthermore, numerous advanced malignant tumor cells harbor intrinsic defects in the STING pathway, thereby creating an ideal environment for OVs replication (13). It should be noted that the complete molecular mechanisms underlying this reverse regulation phenomenon have not yet been fully elucidated. Further exploration of STING downstream effectors related to viral replication can be conducted through proteomics profiling.

Low CAR expression in mouse LLC cells is known to compromise the infectivity of HAdV5 and other adenoviruses that primarily utilize CAR as their receptor (32, 33). In this study, Ad5/3 fiber chimeric OAds were employed, though their infectivity toward LLC cells remained unclear. Consequently, LLC cells were infected with OAd-McGAS, and the results demonstrated efficient secretion of GM-CSF and expression of cGAS, along with observable CPE. Furthermore, the expression of GM-CSF and cGAS was detectable in tumor tissues from LLC syngeneic mice subsequent to i.t. injection of OAd-McGAS. These results indicate that OAd-McGAS can infect LLC cells and drive the expression of target proteins.

The TME exerts a pivotal regulatory role in the therapeutic efficacy of OAds. In this study, we demonstrated that OAd-HcGAS effectively activates the STING pathway in tumor cells under *in vitro* conditions, which in turn elicits IFN-I induction, upregulates the expression of chemokines CXCL10 and CXCL9, and enhances PD-L1 expression. Using the Transwell assay, we verified that chemokines secreted by OAd-HcGAS–infected NCI-H226 cells potently recruit lymphocytes to migrate into Transwell chambers. Notably, significant activation of human T cells and NK cells was observed in an *in vitro* co-culture system established using OAd-infected tumor cells. In C57BL/6N mice bearing LLC syngeneics, substantial infiltration of lymphocytes—including CD8^+^ T lymphocytes, cDCs, and tumor-associated macrophages—was detected within the tumor tissues. Furthermore, CD8+ T cells isolated from these tumor tissues exhibited remarkable cytotoxicity against LLC-EGFP target cells. Collectively, cGAS-armed OAds can reprogram the TME, transforming “cold tumors” into “hot tumors.” Leveraging this characteristic, we optimized anti-tumor efficacy through combination therapy with OAd-HcGAS and PD-L1 blockade.

In C57BL/6N mice, OAds elicited a clear abscopal response in bilateral lung cancer syngeneics. In contrast, no such effect was detected in BALB/c-nu mice bearing bilateral NCI-H226 tumors. These findings suggest that the abscopal activity of OAds is primarily contingent upon the activation of systemic anti-tumor immune responses (34, 35). In this study, significant lymphocyte infiltration was observed in the TME, including CD8^+^ T lymphocytes, cDC cells, and tumor-associated macrophages, on both the treated and untreated sides. The robust cytotoxic capacity exhibited by these infiltrating lymphocytes further supports effective induction of systemic antitumor immunity.

Several limitations of this study should be acknowledged. First, we did not perform direct cytotoxicity and replication assays in normal human cells, which would further support the tumor-selective nature of OAd-HcGAS. Second, while our data demonstrate a correlation between cGAS-STING pathway activation and antitumor efficacy, we have not established a definitive causal relationship. The use of H-151 *in vitro* provides suggestive evidence for STING involvement, but *in vivo* validation using STING-deficient mice, cGAS-knockdown tumor cells, or type I IFN signaling blockade would be required to confirm STING dependency. Third, as the virus encodes two transgenes, GM-CSF and cGAS, we did not systematically evaluate the individual and combined contributions of each transgene to the overall antitumor efficacy. In addition, the GM-CSF encoded by our vector is of human origin, and its biological activity in mice is limited due to the low affinity of the murine GM-CSF receptor for the human cytokine. Nevertheless, the significant antitumor efficacy and CD8^+^ T-cell infiltration observed in immunocompetent C57BL/6 mice suggest that the oncolytic activity and cGAS-STING pathway activation, together with the moderate contribution of human GM-CSF, were sufficient to trigger robust antitumor immune responses. Future studies using a murine GM-CSF-encoding vector or a humanized mouse model, along with normal-cell assays, genetic loss-of-function approaches, and systematic comparison of single- versus dual-transgene constructs, will further elucidate the mechanisms and fully validate the safety and efficacy of this approach.

We engineered oncolytic adenoviruses, OAd-HcGAS and OAd-McGAS, co-expressing cGAS and GM-CSF, which in combination with PD-L1 antibody demonstrated potent antitumor efficacy, offering a novel therapeutic strategy for lung cancer treatment. In subsequent studies, we further optimized these vectors to circumvent the constraint imposed by STING signaling loss and systematically clarified the molecular mechanisms that enable efficient viral replication following STING pathway activation. Particular emphasis was placed on the interactions between virus-encoded immunomodulatory proteins and host antiviral pathways. By delineating the distinct regulatory networks that differentiate OVs from conventional antiviral responses, these findings lay a critical theoretical foundation for the development of next-generation OV-based therapies.

## Materials and methods

### Cell culture

The human squamous lung carcinoma cell line NCI-H226, lung adenocarcinoma cell line A549, colon cancer cell line HCT116, melanoma cell line A2058, prostate cancer cell line PC3, as well as murine Lewis lung carcinoma cell lines LLC and LLC-EGFP were obtained from the Institute of Basic Medical Sciences (IBMS) of the Chinese Academy of Medical Sciences. NCI-H226 cells were cultured in RPMI 1640 medium (EallBio, 03.4007C, China); HCT116 cells in McCoy’s 5A medium (Procell system, PM150710, China); and PC3 cells in Ham’s F-12K medium (Gibco, 21127022, USA). LLC, LLC-EGFP, A549, and A2058 cells were maintained in Dulbecco’s Modified Eagle Medium (HyClone Laboratories, SH3024301, USA). All media were supplemented with 10% fetal bovine serum (Gibco, 15630-080, Australia), 100 IU/mL penicillin, 100 μg/mL streptomycin (EallBio, F240311, China), and 2 mM L-glutamine (Thermo Fisher Scientific, 21051024, USA). All cell lines were maintained in a humidified incubator at 37°C with 5% CO_2_.

### Oncolytic adenovirus preparation

Building on our previously developed hTERT-regulated Ad5/3 chimeric oncolytic adenovirus OAd-Z2, which expresses GM-CSF in a tumor-specific manner, we fused either the human or murine cGAS gene to the E1B55k locus of the OAd-Z2 genome via P2A to generate the hTERT-regulated Ad5/3 chimeric oncolytic adenoviruses OAd-HcGAS and OAd-McGAS, both capable of co-expressing GM-CSF and cGAS. Both OAd-HcGAS and OAd-McGAS were rescued and propagated in HEK 293 cells. Viral titers were measured by Adeno-X Rapid Titer kit (Clontech, Mountain View, USA) after purification using cesium chloride density gradient centrifugation, as previously described.

### Transmission electron microscopy

The purified viruses were adsorbed onto a 200-mesh copper grid for 1 min, followed by staining with phosphotungstic acid for 1 min. The grid was subsequently placed in a storage box and allowed to dry completely before examination. Imaging was performed using a JEM-1400 electron microscope, and images were acquired using an 830.10U3 CCD camera system.

### Viral titer determination

Viral titers were determined using an immunofocus assay. HEK293 cells were seeded in 24−well plates at a density of 2 × 10^5^ cells/well and cultured for 4 h. The virus was serially diluted 10−fold from 10^−3^ to 10^−7^, and 50 µL of each dilution was added to the cells in triplicate. After 48 h of infection, the cells were fixed with pre−chilled 95% ethanol at −20 °C for 30 min, washed with PBS, and blocked with 5% non−fat milk. The cells were sequentially incubated with a primary antibody (mouse anti−adenovirus type 5 serum, 1:1000) and a secondary antibody (HRP−conjugated goat anti−mouse IgG, 1:2000), followed by development with precipitable TMB substrate. Wells containing 10–50 foci per field were counted. The titer was expressed as infectious units (IFU) per milliliter, where one IFU corresponds to one infectious viral particle capable of infecting a cell and expressing the hexon protein. The titer was calculated using the formula: IFU/mL = average number of foci per field × field conversion factor × dilution factor × 20.

### Cytotoxicity assays

The cytotoxic effects of OAd-Z2 and OAd-HcGAS were evaluated using crystal violet and CCK-8 assays across a panel of human cancer cell lines, including NCI-H226, A549, HCT116, A2058, and PC3.

For the crystal violet assay, cells were seeded in 24-well plates at a density of 2×10^5^ cells per well and infected with viruses at MOIs of 0, 0.1, 0.5, 1, 5, and 10. At 96 h post-infection, the cells were fixed with 4% paraformaldehyde for 30 min and stained with a 0.1% crystal violet solution for 10 min. After staining, the wells were gently washed three times with PBS, and images were captured.

For the CCK-8 assay, cells were plated in 96-well plates at 1.5×10^4^ cells per well and infected at MOIs of 0, 0.01, 0.1, 1, 10, and 100. At 24, 48, 72, 96, and 120 h post-infection, 10 µL of CCK-8 solution was added to each well. The absorbance at 450 nm was measured using a microplate reader within 2 h. Cell viability was calculated relative to the mock-infected control cells (set at 100%) using the following formula:


$$\text{Cell viability }\left(\%\text{ control}\right)=\left(\frac{\text{the absorbance of virus infected cells}}{\text{the absorbance of virus non}-\text{infected cells}\;}\right)\times 100\%$$

### Western blotting

After 48 h following infection with OAd-HcGAS, NCI-H226 cells were harvested and lysed for Western blotting analysis. Inhibition of STING activation using the STING antagonist H-151 (MCE, HY-112693, China) was included as a control. For PD-L1 detection, membrane proteins were extracted using a membrane protein extraction kit (Epizyme, PC202, China). Proteins were resolved under reducing conditions (100 mM 2-mercaptoethanol, boiled) on 10% SDS-PAGE gels and transferred to PVDF membranes. The membranes were then incubated overnight at 4 °C with primary antibodies (detailed in the online **Supplementary Materials**), followed by a 1 h incubation at room temperature with fluorescently labeled secondary antibody (1:10000) in the dark. Protein bands were visualized and analyzed using a two-color infrared fluorescence imaging system (ODYSSEY CLX, Li-Cor Biosciences, NE, USA). For analysis of signaling pathway proteins, the membranes were stripped and reprobed with specific antibodies.

### Assessment of cytokine expression by ELISA

For cell culture supernatants, samples were centrifuged at 2,000 × g for 5 min to remove cellular debris. The clarified supernatants were then analyzed using respective ELISA kits according to the manufacturers’ protocols for the detection of human GM-CSF, mouse GM-CSF, human IFN-β, CXCL10, CXCL9, and TNF-α.

For tumor tissue samples, approximately 0.2 g of tissue was homogenized in RIPA lysis buffer, followed by centrifugation at 12,000 rpm for 10 min. The resulting lysates were subsequently assayed for mouse GM-CSF levels using a specific ELISA kit.

### Transwell assay for evaluating the effect of OAd-HcGAS on lymphocyte migration *in vitro*

Cryopreserved human PBMCs (HyCells, 059K002, China) were thawed in a 37 °C water bath and resuspended in RPMI-1640 complete medium supplemented with 10% fetal bovine serum (FBS), anti-CD3 (2 µg/mL), anti-CD28 (2 µg/mL), and IL-2 (10 ng/mL). PBMCs were seeded at 1 × 10^6^ cells/mL in U-bottom 96-well plates and cultured for 4 days. NCI-H226 cells were seeded at 2 × 10^5^ cells/well in 24-well plates overnight. The medium was then replaced with maintenance medium containing 2% FBS, and cells were infected with OAd-Z2 or OAd-HcGAS at an MOI of 20, with PBS as a negative control. After 48 h of infection, culture supernatants were collected and centrifuged at 500 × g for 10 min. Activated PBMCs were harvested, washed, and resuspended in RPMI-1640 containing 0.3% FBS at a concentration of 3 × 10^7^ cells/mL. Then, 100 µL of PBMC suspension was added to the upper Transwell chamber (5.0-µm pore size), and 500 µL of tumor cell supernatant was added to the lower chamber. Following incubation for 2 h at 37 °C, the cells in the lower chamber were collected, mixed, and allowed to settle for 5 min. Cells were photographed in three random fields per well and counted using a hemocytometer.

### Co-culturing of immune cells and NCI-H226 cells in the presence of OAd-Z2 or OAd-HcGAS

For direct *in vitro* co-culture experiments, NCI-H226 cells were plated with PBMCs at a 1:10 ratio in RPMI-1640 medium supplemented with 10% FBS. After 24 h of incubation, the cultures were infected with OAds or OAd-HcGAS at a multiplicity of infection of 10 and incubated for an additional 48 h. The supernatant was collected for LDH detection, and the isolated PBMCs were detected by flow cytometry.

### Detection of PBMC cytotoxicity

The cytotoxicity of PBMCs was estimated by measuring LDH activity in the culture medium using the LDH Cytotoxicity Assay Kit (Beyotime, C0017, China). Briefly, assays were carried out in 96-well plates with a final sample volume of 120 µL/well. The LDH released reagent was added at 10% of the volume of the original culture medium.

### Flow cytometry analysis of P-PBMCs (CD8^+^, CD56^+^, CD69^+^ and IFN-γ^+^)

PBMCs isolated from the co-culture system were transferred into U-bottom 96-well plates and blocked with Human TruStain FcX™ (BioLegend, 422302, USA). CD8-, CD56-, and CD69-conjugated fluorescent antibodies were added, and cells were incubated in the dark at 4 °C for 30 min. Following a single wash, cells were fixed with fixation buffer for 20 min in the dark. Fixed cells were then resuspended in diluted Intracellular Staining Permeabilization Wash Buffer (BioLegend, 421002, USA), and centrifuged at 350 × g for 5–10 min; this step was repeated twice. Finally, IFN-γ-conjugated fluorescent antibodies were added and incubated in the dark at 4 °C for 30 min.

### Animal studies

Specific pathogen-free female C57BL/6 mice (aged 6–8 weeks) and BALB/c-nu immunodeficient mice (aged 6–8 weeks) were purchased from Vital River Laboratory Animal Technology Ltd. (Beijing, China). All mice were maintained under SPF conditions. All animal experiments were approved by the Animal Welfare and Research Ethics Committee of Beijing Laboratory Animal Research Center (BLARC-LAWER-202407009).

BALB/c-nu immunodeficient mice (aged 6–8 weeks) were subcutaneously inoculated bilaterally in the flanks with NCI-H226 cells (5 × 10^6^ cells/mouse). When tumor volumes reached approximately 50–100 mm³, mice were stratified by tumor volume and randomly assigned to treatment groups. For the therapeutic regimen, one side of the bilateral tumors was intratumorally (i.t.) injected with OAds (1 × 10^8^ PFU/tumor) every 2 days for a total of 6 doses, while the contralateral side remained untreated.

C57BL/6 mice (aged 6–8 weeks) were subcutaneously inoculated bilaterally in the flanks with LLC cells (5 × 10^6^ cells/mouse). After tumor volumes reached approximately 50–100 mm³, mice were stratified by tumor volume and randomly assigned to treatment groups. For the therapeutic regimen, one side of the bilateral tumors was intratumorally injected with OAds (1 × 10^8^ PFU/tumor) every 2 days for a total of 5 doses, while the other side was left untreated.

For the combination therapy experiment, C57BL/6 mice bearing bilateral LLC tumors were treated with OAd-McGAS (1 × 10^8^ PFU/tumor, i.t., every 2 days for 2 doses) in combination with PD-L1 antibody (5 mg/kg, intraperitoneally, every 2 days for 4 doses).

Subcutaneous tumor dimensions were measured every 2 days using a digital vernier caliper. The tumor volume (mm^3^) was calculated as L × W^2^/2, where L and W represent tumor length and width, respectively. Once tumor volumes reached approximately 50–100 mm³, mice were randomly assigned to treatment groups and received intratumoral (i.t.) injections. The tumor growth inhibition rate (TGI) was calculated based on relative changes in tumor volume using the following formula:


$$\text{TGI}=\left[1-\frac{{\text{RTV}}_{\text{treatment}}}{{\text{RTV}}_{\text{control}}}\right]\times 100\%,$$

where relative tumor volume (RTV) is defined as V_t_/V_0_, with V_t_ representing the mean tumor volume at endpoint (Day 10) and V_0_ representing the mean tumor volume at baseline (Day 0).

### Multicolor flow cytometry

Tumor tissues or spleen were collected from mice, minced, and digested in RPMI-1640 medium containing 0.1 mg/mL collagenase I (Gibco, 17018029, USA), 1 mg/mL collagenase IV (Gibco, 17104019, USA) and 200 U/mL DNase I at 37 °C for 1 h. The resulting cell suspension was passed through a 70-μm cell filter (Beyotime, FSTR070, China), washed with PBS, and resuspended in cell staining buffer (BioLegend, 420201, USA). Single-cell suspensions were first blocked with TruStain FcX™ (BioLegend, 101320, USA) for 10 minutes at 4 °C, followed by staining with a fluorescent antibody cocktail (Supplemental Materials) for 40 minutes at 4 °C. Prior to surface staining, cell viability was assessed using the Zombie NIR™ Fixable Viability Kit (BioLegend, 423106, USA) by incubating cells at room temperature in the dark for 15 minutes. Samples were acquired on a FACSCanto II system or FACSFortessa system (BD Biosciences, CA, USA), and data were analyzed using FlowJo software (BD Biosciences, CA, USA).

### Killing capacity of TILs

CD4^+^ and CD8^+^ TILs were isolated from both treated and untreated tumor tissues using CD4/CD8 (TIL) MicroBeads (Miltenyi, 130-116-480, German). TILs were co-cultured with LLC-EGFP target cells at different effector-to-target ratios (0:1, 1:1, and 5:1) in a 37 °C, 5% CO_2_ incubator for 24 h. Surviving target cells expressing green fluorescence were visualized and captured using a fluorescence microscope. Relative fluorescence units (RFU) were measured at an excitation wavelength of 479 nm and an emission wavelength of 517 nm using a microplate reader.

### Quantitative PCR

Total DNA was extracted from tumor tissues using a Blood/Cell/Tissue DNA Extraction Kit (Tiangen, DP304-03, China). qPCR was performed using a fluorescence-quantitative PCR kit (Tiangen, FP205-02, China) on a q225 system (KUBO technology, Beijing, China), according to the manufacturer’s instructions. The primer sequences targeting the viral hexon gene were as follows: forward, 5′-GGTGGCCATTACCTTTGACTCTTC-3′; reverse, 5′-CCACCTGTTGGTAGTCCTTGTATTTAGTATCATC-3′.

### Hematoxylin and eosin staining, immunohistochemistry, and immunofluorescence

Liver tissues were processed for H&E staining following standard protocols and analyzed under a light microscope (Olympus, Tokyo, Japan). For all staining procedures, tissue sections were deparaffinized, rehydrated, and washed in 1% PBST. For IHC, the sections were soaked in 3% hydrogen peroxide to block endogenous peroxidase activity and incubated with primary antibodies overnight at 4 °C. The sections were then incubated with HRP-linked anti-rabbit antibodies for 20 min, developed with diaminobenzidine substrate, and counterstained with hematoxylin. For IF, tissue sections were incubated with primary antibodies overnight at 4 °C, followed by fluorescently labeled secondary antibodies for 40 min the next day, and then counterstained with DAPI for 8 min. Images were acquired by fluorescence microscopy (Olympus, Tokyo, Japan) and analyzed using ImageJ software. A complete list of primary antibodies is provided in the **Supplementary Materials**. The secondary antibodies used were as follows: for IHC, a rabbit two-step detection kit (ZSGB-Bio, PV-6001, China); for IF, Anti-Mouse Cy3 (Beyotime, P0193, China) and Anti-Rabbit FITC (Beyotime, P0186, China).

For ImageJ quantification, all images were analyzed using a consistent thresholding method. For each group, 3 tumors were examined, with 1 section per tumor and 5 randomly selected fields per section. All quantitative analyses of immunofluorescence and immunohistochemistry images were performed in a blinded manner, with the operator unaware of the experimental group assignments.

### Statistical analysis

Statistical analysis was performed using the SPSS software version 21 (Chicago, IL, USA), and one-way analysis of variance with SNK-q post-test was used to compare the means of multiple groups. *P* < 0.05 was considered significant. The data were presented as mean ± SD.

## Data Availability

The datasets presented in this study can be found in online repositories. The names of the repository/repositories and accession number(s) can be found in the article/**Supplementary Material**.
