# Supplementary material for: Oncolytic adenovirus armed with cGAS activates STING pathway and enhances antitumor immunity in lung cancer with superior combined efficacy of PD-L1 therapy
Source: Front Immunol. 2026 Jul 23;17:1904206. doi: 10.3389/fimmu.2026.1904206 (PMC13442440; doi:10.3389/fimmu.2026.1904206)
Supplement: Supplementary file 1 [file DataSheet1.pdf]

Supplemental Figure legends:

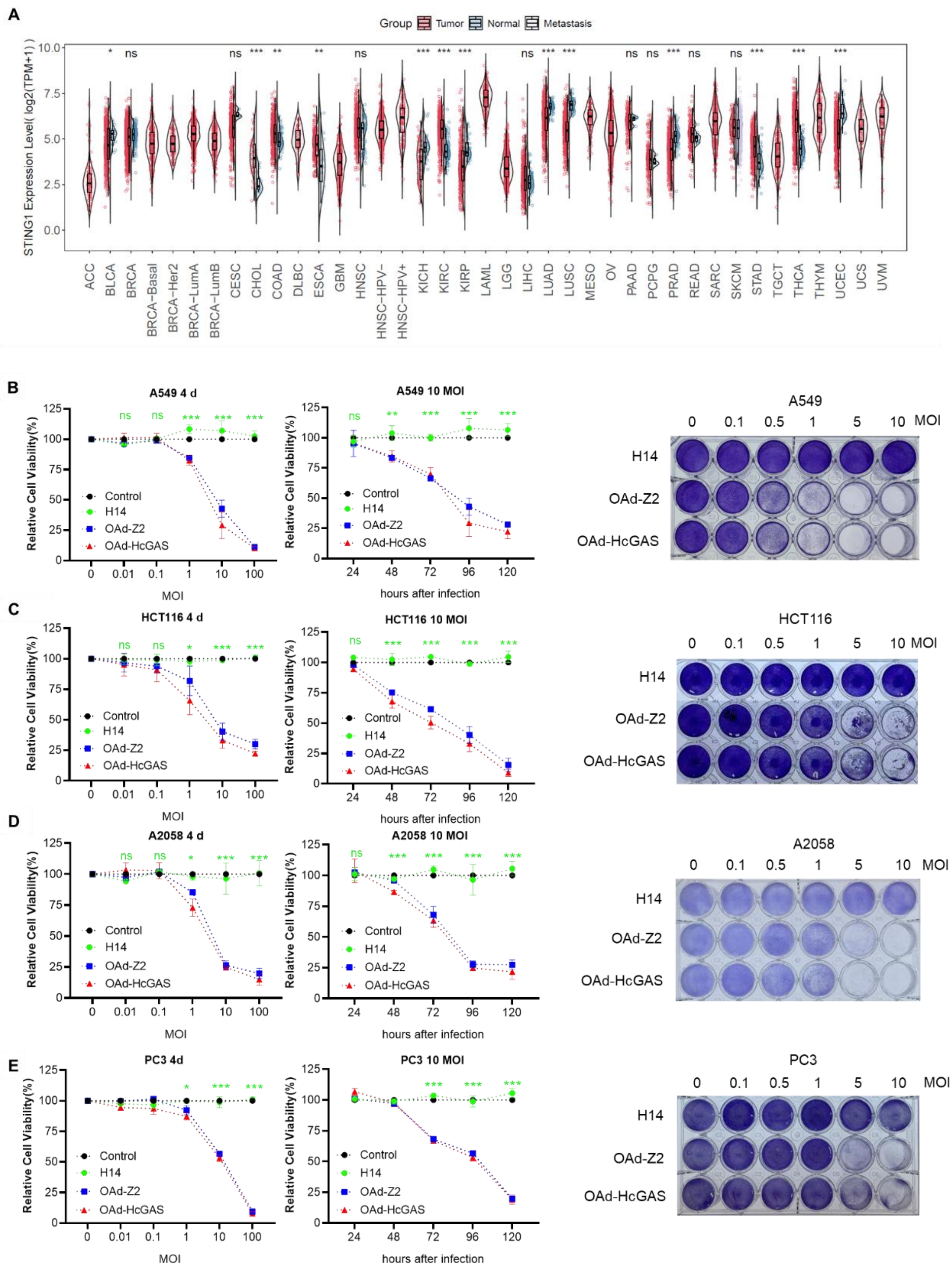

Supplemental Figure 1. OAd-HcGAS effectively induces cell death in tumor cells *in vitro*. (A) STING1 expression between tumor and normal samples analyzed by TIMER3.0 (https://compbio.cn/timer3/). The viability of A549 **(B)**, HCT116 **(C)**, A2058 **(D)**, and PC3 **(E)** cells was evaluated using the CCK-8 assay at 24, 48, 72, 96, and 120-hours post-infection with OAds at varying MOIs, as well as at different time points following

infection with a fixed dose of 10 MOIs. Qualitative assessment of the cytocidal effect was conducted using the crystal violet assay at 96 hours post-infection across different MOI levels. Data are presented as mean ±SD. $^{*}p < 0.05$, $^{**}p < 0.01$, $^{***}p < 0.001$; ns: no significant difference.

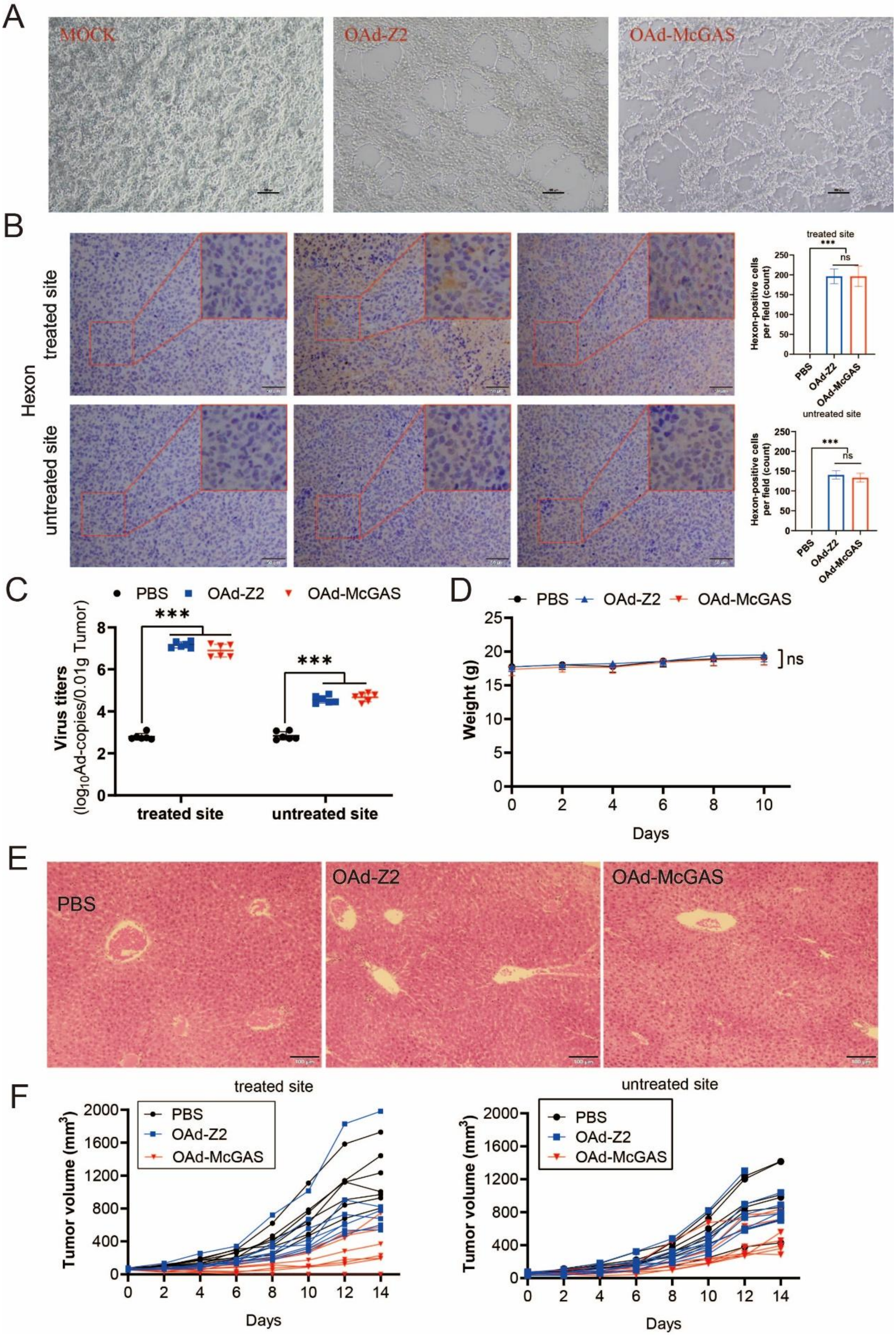

Supplemental Figure 2. OAds can infect LLC cells and tumor tissues. (A) Cytopathic effects of oncolytic adenovirus in LLC cells (100 μm); **(B)** Representative adenovirus Hexon protein immunohistochemistry (50 μm) and quantification of Hexon+ cells in three

random fields from treated and untreated tumor tissues; **(C)** The viral titers in treated and untreated tumor tissues detected by qPCR; **(D)** Mouse weight. **(E)** HE staining of mouse liver. (F) Data were shown as $\overline{X} \pm SD$, $***$ p $<$ 0.001, ns: no significant difference.

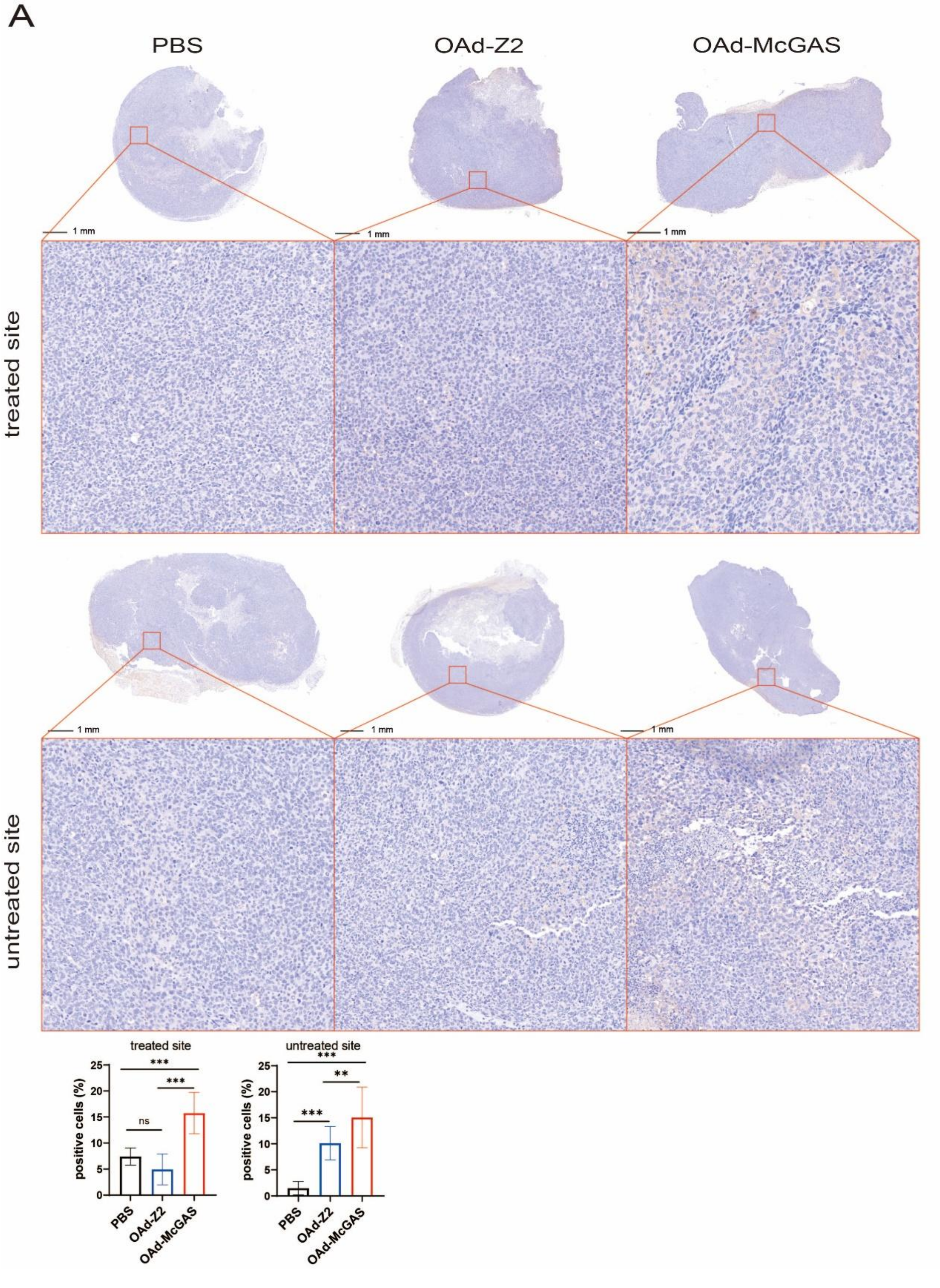

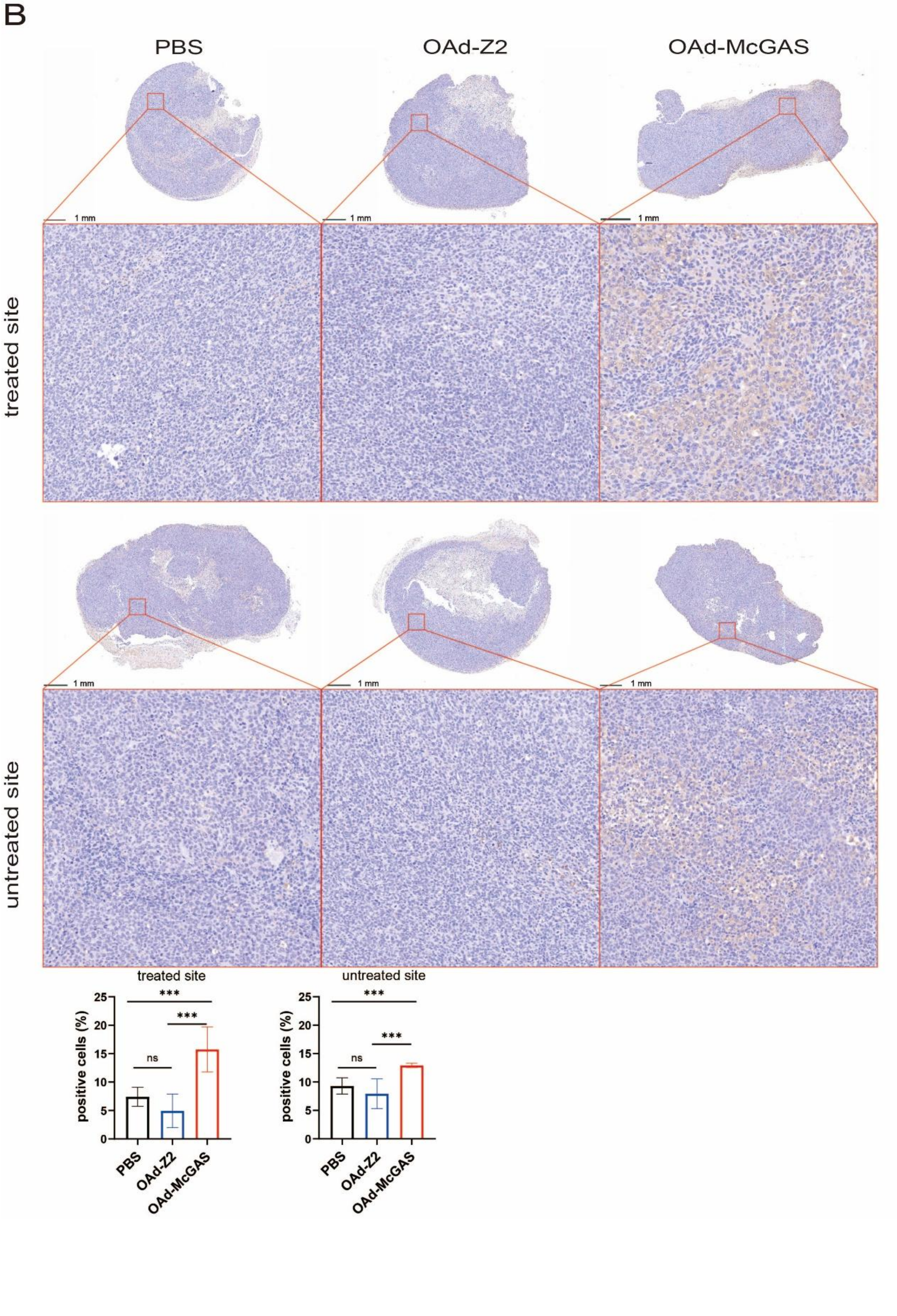
B
PBS
OAd-Z2
OAd-McGAS
1 mm
treated site
untreated site
treated site
untreated site
positive cells (%)
25
20
15
10
5
0
***
***
ns
PBS
OAd-Z2
OAd-McGAS

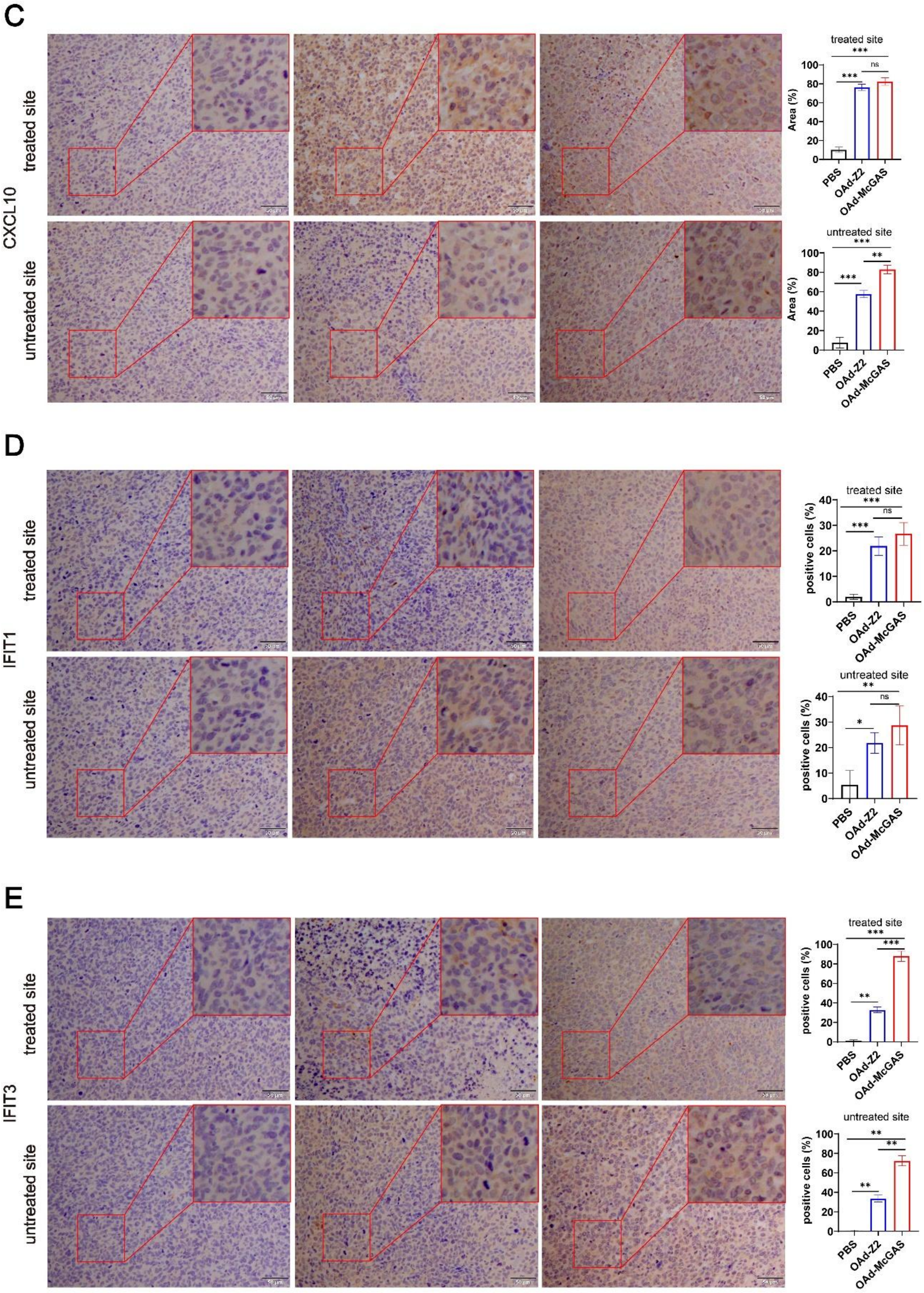
C
CXCL10
treated site
untreated site
treated site
Area (%)
100
80
60
40
20
0
***
***
ns
PBS
OAd-Z2
OAd-McGAS
untreated site
Area (%)
***
***
**
D
IFIT1
treated site
untreated site
treated site
positive cells (%)
40
30
20
10
0
***
***
ns
untreated site
positive cells (%)
**
*
ns
E
IFIT3
treated site
untreated site
treated site
positive cells (%)
***
***
**
untreated site
positive cells (%)
**
**
**
50 μm

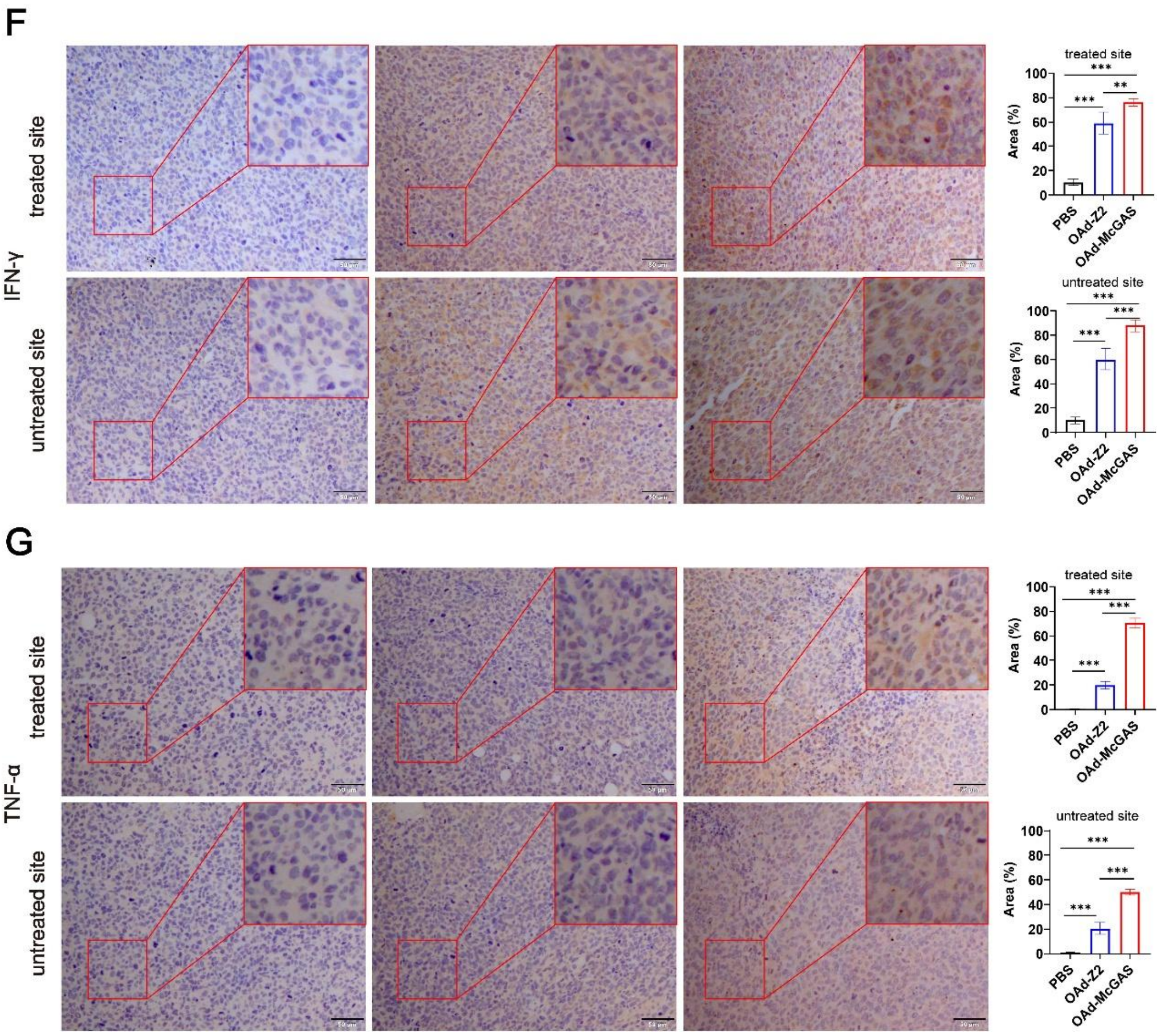

Supplemental Figure 3. OAd-McGAS therapy can effectively activate STING pathway. **(A)** TBK1 situation (1 mm) and the proportion of TBK1 positive areas in the five random visual fields were statistically analyzed; **(B)** IRF3 situation (1 mm) and the proportion of IRF3 positive areas in the five random visual fields were statistically analyzed; **(C)** CXCL10 situation (50 μm) and the proportion of CXCL10 positive areas in the three random visual fields were statistically analyzed; **(D)** IFIT1 status (50 μm) and statistics of the proportion of IFIT1 positive cells in three random fields; **(E)** IFIT3 status (50 μm) and statistics of the proportion of IFIT3 positive cells in three random fields; **(F)** IFN-γ situation (50 μm) and statistics of the proportion of IFN-γ positive regions in the three random visual fields; **(G)** TNF-α situation (50 μm) and were used to statistically analyze the proportion of TNF-α positive regions in the three random visual fields.

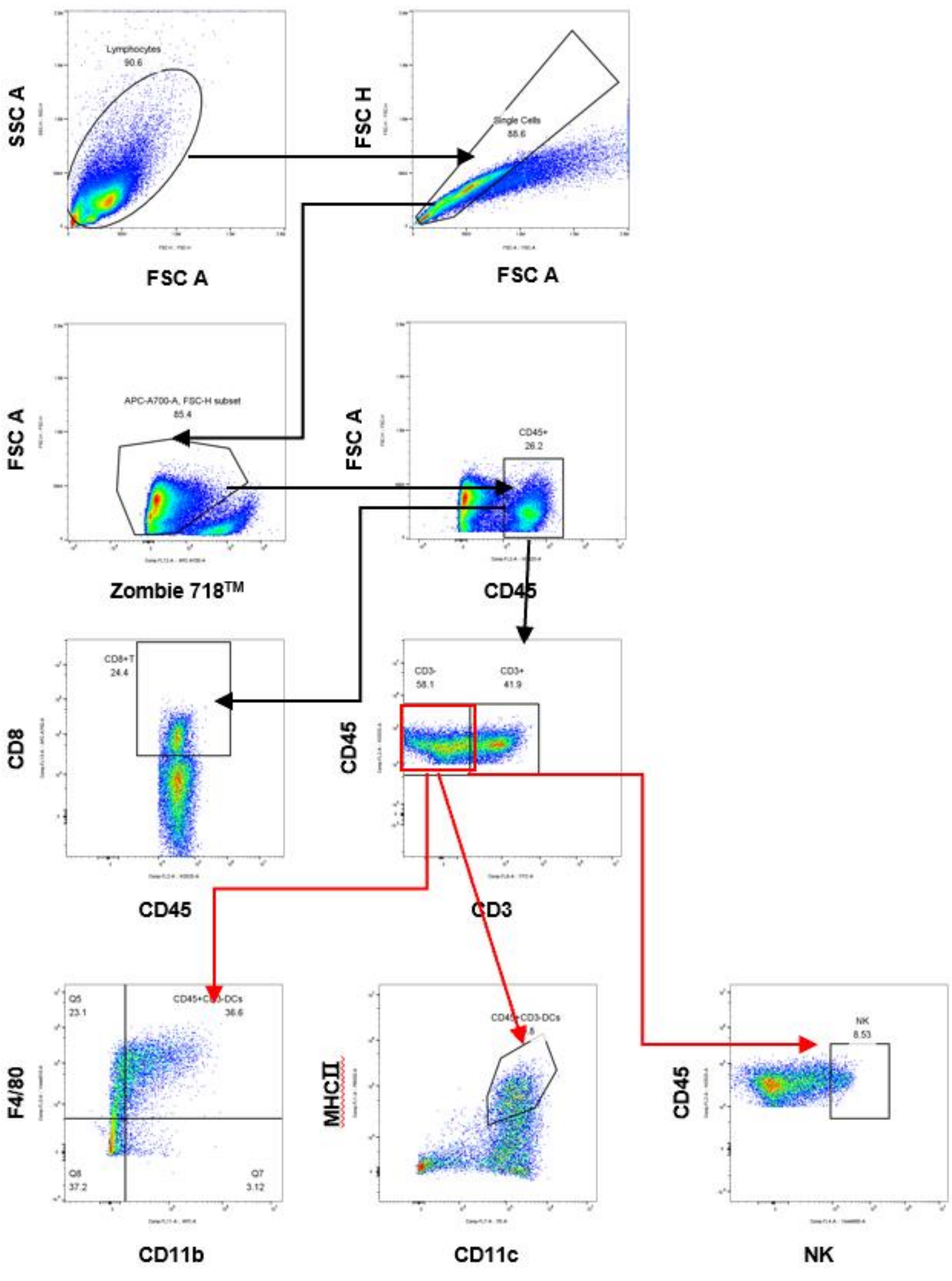

Lymphocytes
90.6
SSC A
FSC A
Single Cells
88.6
FSC H
FSC A
APC-A700-A, FSC-H subset
85.4
FSC A
Zombie 718TM
CD45+
26.2
FSC A
CD45
CD8+T
24.4
CD8
CD45
CD3-
58.1
CD3+
41.9
CD45
CD3
Q5
23.1
Q8
37.2
Q7
3.12
F4/80
CD11b
CD45+CD3-DCs
MHCII
CD11c
NK
8.53
CD45
NK

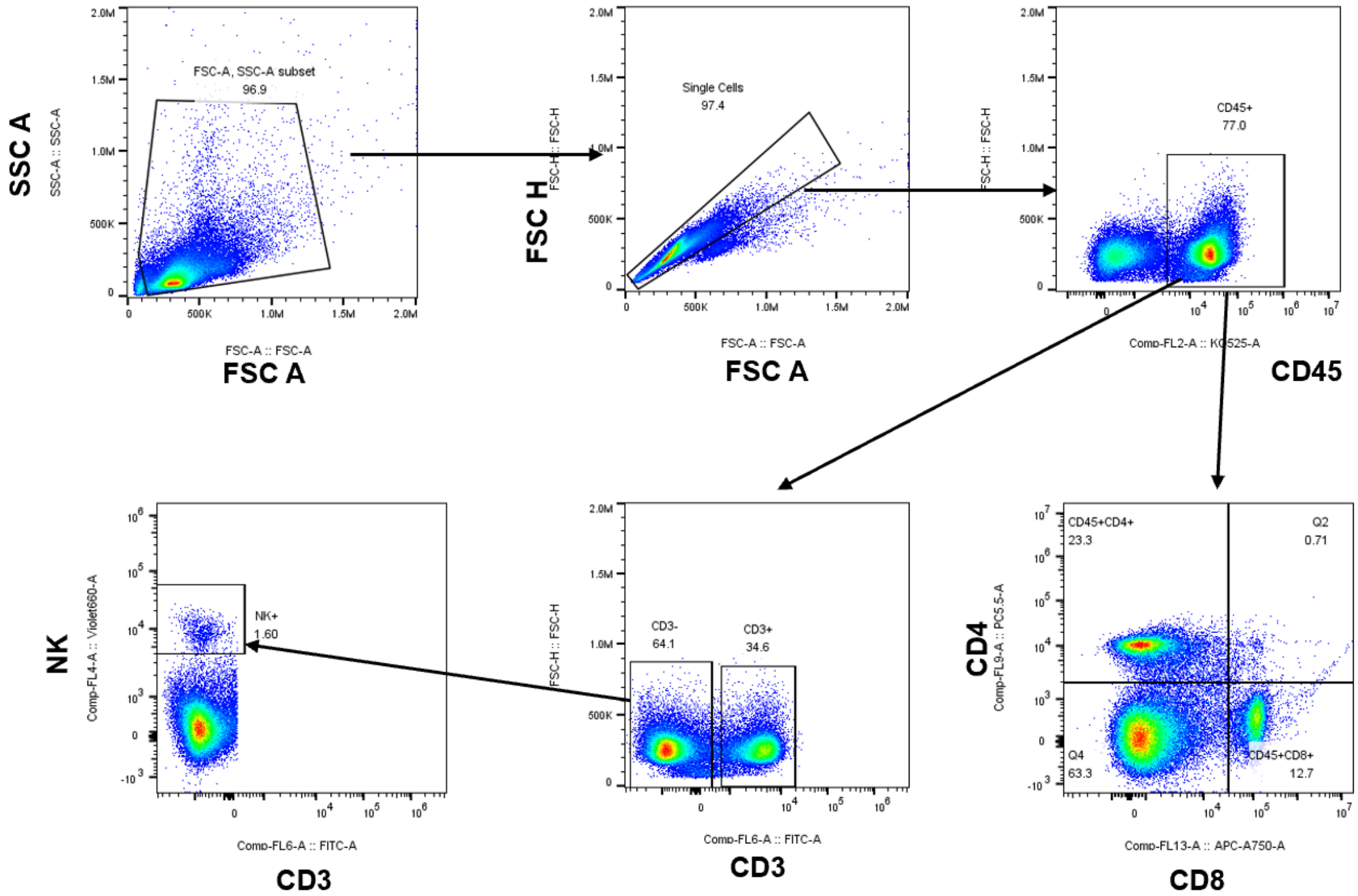

- **Supplemental Figure 4. Flow Cytometry Diagram**

Supplemental Materials

Antibodies of IHC/IF

The antibodies used in IHC and IF included anti-Ki-67 (bs-23013R, Bioss, Beijing, CN), anti-Hexon (NB600-1386, Novus, USA), anti-CD8 (bs-0648R; Bioss, Beijing, CN), anti-CXCL10 (abs135937, Absin, Beijing, CN), anti-IFN-γ (abs135565, Absin, Beijing, CN), anti-TNF-α (AF8208, Beyotime, Beijing, CN), anti-pIRF3 (29528-1-AP, Proteintech, USA), anti-pTBK1 (5483, CST, USA), anti-IFIT1 (23247-1-AP, Proteintech, USA) and anti-IFIT3 (15201-1-AP, Proteintech, USA), Rabbit anti-TBK1 antibody (Cell Signaling Technology, 3504, USA), Rabbit anti-IRF3 antibody (proteintech, 11312-1-AP, CN).

Antibodies of Western blotting

The cell lysate was then subjected to immunoblotting with Rabbit anti-phospho-STING antibody (Cell Signaling Technology, 72971, USA), Rabbit anti-phospho-TBK1 antibody (Cell Signaling Technology, 5483, USA),Rabbit anti-TBK1 antibody (Cell Signaling Technology, 3504, USA), Rabbit anti-phospho-IRF3 antibody (Cell Signaling Technology, 29047, USA), Rabbit anti-IRF3 antibody (proteintech, 11312-1-AP, CN), anti-β-actin Rabbit

(Abclonal, AC026, CN).

Antibodies of flow cytometry

The antibody panel included: Brilliant Violet 510™ anti-mouse CD45 (Biolegend, 103138, USA), FITC anti-mouse CD3 (Biolegend, 100204, USA), PerCP/Cyanine5.5 anti-mouse CD8a (BioLegend, 100734, USA), APC anti-mouse CD11c (BioLegend, 117310, USA), PE anti-mouse/human CD11b (BioLegend, 101208, USA), Brilliant Violet 421™ anti-mouse F4/80 (BioLegend, 123132, USA), and Brilliant Violet 605™ anti-mouse I-A/I-E (BioLegend, 107639, USA), Brilliant Violet 650™ anti-mouse NK-1.1 (BioLegend, 108736, USA).
